# Wnt Pathway Activation Increases Hypoxia Tolerance during Development

**DOI:** 10.1371/journal.pone.0103292

**Published:** 2014-08-05

**Authors:** Merril Gersten, Dan Zhou, Priti Azad, Gabriel G. Haddad, Shankar Subramaniam

**Affiliations:** 1 Bioinformatics and Systems Biology Graduate Program, University of California San Diego, La Jolla, California, United States of America; 2 Department of Pediatrics, University of California San Diego, La Jolla, California, United States of America; 3 Department of Neuroscience, University of California San Diego, La Jolla, California, United States of America; 4 Rady Children's Hospital, San Diego, California, United States of America; 5 Department of Bioengineering, University of California San Diego, La Jolla, California, United States of America; 6 Departments of Cellular and Molecular Medicine, Chemistry and Biochemistry and Nanoengineering, University of California San Diego, La Jolla, California, United States of America; University of Dayton, United States of America

## Abstract

Adaptation to hypoxia, defined as a condition of inadequate oxygen supply, has enabled humans to successfully colonize high altitude regions. The mechanisms attempted by organisms to cope with short-term hypoxia include increased ATP production via anaerobic respiration and stabilization of Hypoxia Inducible Factor 1α (HIF-1α). However, less is known about the means through which populations adapt to chronic hypoxia during the process of development within a life time or over generations. Here we show that signaling via the highly conserved Wnt pathway impacts the ability of *Drosophila melanogaster* to complete its life cycle under hypoxia. We identify this pathway through analyses of genome sequencing and gene expression of a *Drosophila melanogaster* population adapted over >180 generations to tolerate a concentration of 3.5–4% O_2_ in air. We then show that genetic activation of the Wnt canonical pathway leads to increased rates of adult eclosion in low O_2_. Our results indicate that a previously unsuspected major developmental pathway, Wnt, plays a significant role in hypoxia tolerance.

## Introduction

Hypoxia plays a key pathogenic role in the outcome of a variety of pathologic conditions, including airway obstruction, stroke, and myocardial infarction, and in the increased radio- and chemoresistance of solid tumors [1], [2]. All metazoan organisms require oxygen for growth, development and maintenance, although they vary with respect to the degree and duration of hypoxia that can be tolerated, and may differ in some of the adaptive responses employed to survive hypoxic challenge [3], [4]. Understanding transcriptional and signaling mechanisms that allow more hypoxia-tolerant organisms to thrive chronically at lower oxygen tension may enable us to decipher the mechanisms underlying hypoxia-tolerance observed in many tumors, as well as identify novel approaches for treating disorders in which acute or chronic hypoxia contributes to long-term morbidity and mortality.

We used *D. melanogaster* in a long-term selection experiment, starting with a pool of 27 isogenic lines, to generate a population of flies that is able to reproduce and thrive at 4% O_2_, a level lethal to the parental lines [5], [6], [7]. Starting at 8% O_2_, levels were reduced in a stepwise manner in triplicate chambers with tolerance to 5% O_2_ achieved by generation 13. Three control populations were maintained in parallel in chambers with room air. Phenotypic evaluation of adult hypoxia-adapted flies (AF) at generation 18 revealed the following significant differences from control flies: smaller body size and weight, shortened recovery time from anoxic stupor, and a lesser reduction of O_2_ consumption rate in 3% O_2_; mean lifespan in normoxia was unchanged [6], [7]. Transcriptomic analysis revealed down-regulated metabolic genes and up-regulated Notch and Toll/Imd pathways in larva AF, and significantly fewer gene expression differences in adult AF [7]. Subsequently, the 5% O_2_-maintained populations experienced a bottleneck (during which attempts to further reduce O_2_ failed) before achieving 4% O_2_ tolerance at generation 32, suggesting that genetic mutation and/or selection of favorable alleles, associated with a contraction of genetic variation, may have been required to achieve tolerance to this lower level of O_2_. A genetic and/or epigenetic role in hypoxia tolerance of the AF was also suspected based on the ability of a subset of AF that had been reverted to normoxia for several generations to successfully complete development when returned to a 4%-O_2_ environment [7].

To identify genetic mechanisms underlying the AF adaptation, we re-sequenced pools of control and AF flies that had been under hypoxia selection for 180 generations and adapted to 4% O_2_. We focused our analysis on determining whether genetic selection contributed to our earlier observation in 5% O_2_-adapted flies [7], that Notch pathway activation is one of the factors contributing to hypoxia tolerance [5]. We utilized both a coarse-grained method, which identified 188 genes in 24 hypoxia-selected regions comprising 1.5 mbp, and a fine-grained approach that identified genome-wide high-confidence allelic differences between control and hypoxia-adapted flies. Both analyses identified several genes encoding or regulating the Notch pathway. Here we carry out a comprehensive analysis to determine whether other pathways and processes were selected for by long term O_2_ deprivation. Our aim is to possibly discover hypoxia tolerance-promoting mechanisms which might help explain the bottleneck encountered in achieving 4%-O_2_ tolerance. Existence of such additional mechanisms is also suggested by evidence of Notch cross-talk with other signaling/developmental pathways detected in a network constructed using a set of high-confidence *D. melanogaster* functional interactions [8] and genes identified in the genomic analysis to differ between control and AF (Figure S1). We propose that the polymorphisms identified in the adapted flies were selected for over time from within the initial population variation because of their contribution to an evolving phenotype better suited to a low pO_2_ environment. The presence of multiple polymorphic loci within a given gene and pathway is expected to reflect incremental improvement and arguably compensatory changes to the phenotype, as well as some element of passenger polymorphisms. Therefore identification of many genes, within a particular process or pathway, bearing an excess of polymorphisms suggests that adaptation to hypoxia specifically affects the process or pathway in question. Our extensive analysis of the AF resequencing data was carried out in the context of a new transcriptomic analysis performed on several developmental stages of similarly adapted (4% O_2_-tolerant) AF that are either maintained in hypoxia or reverted to normoxia to identify the impact of oxygen deprivation on gene expression in AF. Here we show that the Wnt signaling pathway is a key factor involved in adaptation to hypoxia during development.

## Results

### Distribution of hypoxia-tolerance polymorphisms across the genome

**Figure 1:**
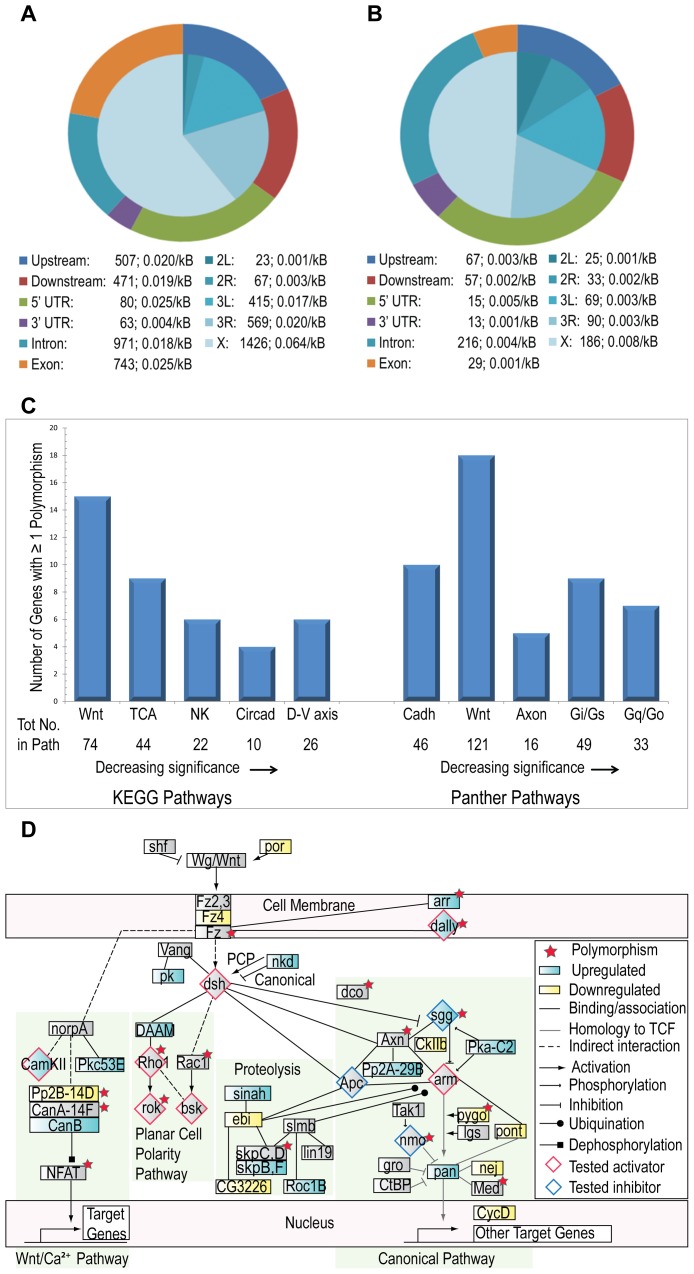
Polymorphisms identified in flies adapted to 4% O_2_ hypoxia. (A,B) Chromosome and gene region distributions of SNPs (A) or indels (B) were normalized to the size of (euchromatin) X chromosome or number of total intron loci, respectively. The legends specify the actual number of polymorphisms detected and polymorphisms/kB (euchromatin) chromosome or gene region. Not included are 14 SNPs that map to XHet, YHet or U/Uextra and 2 indels that map to U and Chromosome 4. (C) DAVID [11], [12]-identified KEGG [14], [15] and Panther [16] pathway enrichment of polymorphisms. The number of pathway genes with ≥1 polymorphism is plotted and the total number of pathway genes is indicated below each pathway name (TCA = Citrate, NK = NK cell mediated cytotoxicity, Circad = Circadian, D-V axis = Dorso-ventral axis formation, Cadh = Cadherin, Axon = Axon guidance (semaphorins), Gi/Gs = Gi/Gs mediated signaling, Gq/Go = Gq/Go mediated signaling). (D) The Wnt pathway was adapted from KEGG [14], [15] with additional interactors added. Genes with one or more fixed polymorphisms are indicated with a red star. Genes differentially expressed in post-eclosion AF maintained in 4% O_2_ are shaded in cyan (up-regulated) or yellow (down-regulated). Wnt pathway genes tested in genetic crosses are diamond-shaped; pathway activators are indicated by a red border and pathway inhibitors by a blue border.

The sequenced populations included one control (C) and two AF (H1, H2) samples with each sample consisting of 200 pooled genotypes. Assuming that emergence of the AF from the 3.5–4% O_2_ reproductive bottleneck implied a majority of AF had acquired a minimum set of required genetic changes, we employed a fine-grained analysis approach to identify genome-wide candidate genes. Specifically, we looked for polymorphisms fixed (≥90%) in H1 and H2 and rare (≤10%) in C at high-quality loci, defined in terms of coverage (≥10X) and Maq [9] reported base quality (≥20) and best read quality (≥40). Figure S2 provides an overview of the methodology employed. Approximately 50% of the euchromatin portion of the five major gene-bearing chromosomes (X, 2L, 2R, 3L, 3R; range 45.4–53.4%) met these criteria in the C dataset as well as in both H1 and H2 datasets and were analyzed for SNPs and small indels. A total of 2514 SNPs and 405 small indels distinguished H1 and H2 from C, of which 1940 SNPs and 283 indels were mapped to a total of 1072 FlyBase [10] extended genes (gene plus 2 kB up- and downstream; Table S1A. Tables of all SNPs and indels mapped to FlyBase genes are available upon request). Applying the same SNP selection criteria to the analyzable loci, we detected only a single locus fixed in one H population and rare in the other, for an estimated FDR of 4.0×10^−4^. Polymorphisms were identified in all gene regions (Figure 1A,B), though relatively fewer mapped to 3′UTR (SNPs, indels), and exons (indels) after normalizing for gene region size. Approximately half the indels and nearly two-thirds of SNPs mapped to the X chromosome after normalization for (euchromatin) chromosome size (Figure 1A, B). At all these polymorphic loci, the H1 and H2 sequences matched each other; this suggests that selection rather than mutation dominated during adaptation to hypoxia.

### Hypoxia-tolerance polymorphisms are enriched for Wnt pathway genes

Functional enrichment analysis is a widely used approach for identifying biologically meaningful processes from high-throughput datasets. Many tools exist, and may be categorized [11] based on enrichment (i) of pre-selected genes that differ between experimental and control samples (SEA), (ii) by gene ranking of all assayed genes (GSEA), and (iii) through an extension of SEA that considers relationships between different annotation terms. Using DAVID [11], [12], a commonly employed annotation enrichment tool, analysis limited to the 99 genes containing non-synonymous coding region polymorphisms was unrevealing. However, analysis of genes with one or more polymorphisms across the extended gene region revealed most top-scoring GO-BP annotations to relate to development and morphogenesis. Lower scoring annotations included 11 genes related to oxidative phosphorylation, 45 genes related to oogenesis and 52 to cell cycle, including ATR homolog, mei-41, with 26 SNPs, which regulates a meiotic checkpoint during Drosophila oogenesis [13] (Table S1B). To account for effects of gene length on annotation enrichment, we calculated the expected (mean) frequency of polymorphisms across all *D. melanogaster* extended genes (µ = 0.024 polymorphisms/kB). Reanalysis of GO-BP annotation using only genes with > = 2 µ (N = 922) or > = 3 µ (N = 869) polymorphism/kB again identified many significant terms related to development and morphogenesis. Pathway analysis of genes with ≥1 polymorphism revealed the Wnt signaling pathway to be at or near the top of both KEGG [14], [15] (N = 15, p = 0.002) and Panther [16] (N = 18, p = 0.055) reported pathways (Figure 1C, Table S1C), a striking result, despite the borderline significance for the Panther pathway. The lesser enrichment significance of the Panther Wnt pathway may reflect inclusion of some genes peripheral to Wnt signaling per se, as Panther focuses on vertebrate pathways and infers shared roles among protein subfamily members, which the developers note may lead to over- or under-prediction for non-vertebrates [17]. We further confirmed KEGG Wnt-pathway enrichment by calculating the hypergeometric p-value for polymorphism enrichment (p = 4.56e–06; see Methods). Additional Wnt-pathway related genes were identified by comparing the list of polymorphic genes with (i) the set of *D. melanogaster* genes assigned a Wnt association by Gene Ontology [18], which added four genes, and (ii) the set of putative canonical Wnt-pathway regulatory genes identified by DasGupta in a genomic RNAi screen [19]. Of the 238 putative regulators identified in the latter screen, 207 were mapped to a total of 212 FlyBase genes, adding another 29 Wnt-related genes for a total of 56 genes among those identified as containing a polymorphism, The overlap between the polymorphic gene set and the DasGupta set was significant, with hypergeometric p-value  = 4.51E–04.

Tables S2A and S2B provide information regarding all SNPs and indels detected in Wnt pathway-associated genes and Table S2C summarizes data on coding region polymorphisms. Table S2D summarizes the number of polymorphisms observed for the 56 Wnt pathway-associated genes and their association (when known) with Wnt signaling. Although the majority of genes (38 of 56) had three or fewer polymorphisms in their analyzable euchromatin regions, four genes had ten or more polymorphisms: *sima* (19), *Smr* (15), *rok* (18) and *hang* (50). When adjusted for extended gene size (gene plus 21KB up- and down-stream), seventeen genes had ≥10 times the expected frequency (µ) of polymorphisms, and five genes had ≥0.9 polymorphisms/KB, or nearly 40 times the expected frequency: *Cby* (1.26), *CG7837* (0.92), *hang* (2.68), *Pp2B-14D* (0.93), and *rok* (1.10). Eighteen genes had one or more coding region SNPs, of which four genes had one or more non-synonymous SNPs: *CG11873* (3 NS), *sima* (1 NS), *pygo* (1 NS) and *hang* (2 NS). Although the majority of coding region SNPs were synonymous, there is recent evidence that a substantial percent of synonymous sites in *D. melanogaster* may evolve under strong purifying selections [20]. The identification of many genes within the Wnt pathway with a frequency of polymorphisms far in excess of the expected frequency suggests that adaptation to hypoxia specifically affects the Wnt signaling pathway.

### Differential gene expression is observed predominantly early after eclosion and requires a hypoxic environment

**Figure 2:**
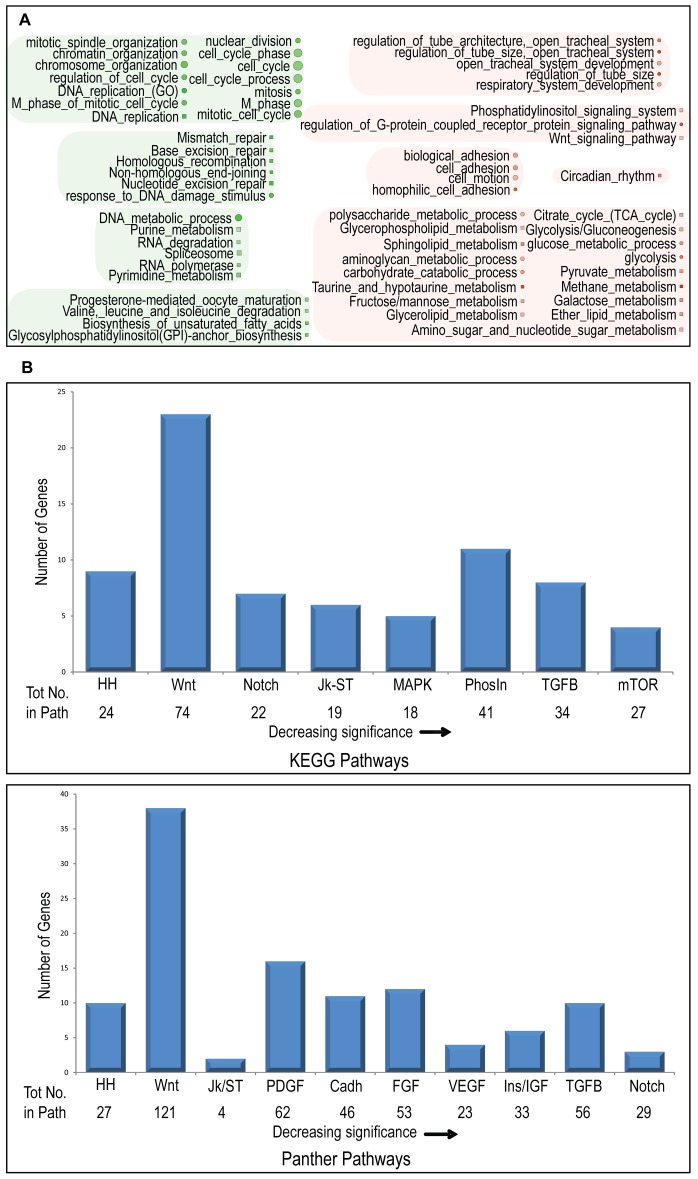
Functional analysis of post-eclosion HR differential expression. (A) The top-scoring KEGG (square) and GO-BP (circle) annotations for up- and down-regulated genes are shown. Down-regulated genes are colored green and are highly enriched for DNA replication, cell cycle and DNA repair. Up-regulated genes are colored red and relate primarily to development and metabolism. In both cases, more intensive color indicates greater fold-enrichment [ranges: (−1.36)–(−4.61); 1.57–4.84] and size reflects the number of genes identified (4–195). (B) DAVID [11], [12]-identified KEGG [14], [15] and Panther [16] signaling pathway enrichment of genes differentially expressed in post-eclosion AF. The number of differentially expressed pathway genes is plotted and the total number of pathway genes is indicated below each pathway name (HH = Hedgehog, Jk-ST = Jak-STAT, PhosIn = Phosphatidylinositol, TGFB = TGF-beta, Cadh = Cadherin, Ins/IGF = Insulin/IGF).

To determine whether there was physiologic evidence for Wnt pathway involvement, during development, in adaptation to 3.5–4% O_2_, we analyzed gene expression in 3^rd^ instar larvae, 1–3 hr post-eclosion flies and 7–9 d adults obtained from three populations: C, AF (“H”), and AF maintained at 21% O_2_ for five generations (“HR”). We included the latter population to investigate the relative contributions of genetics versus environment (hypoxia) to expression differences between C and AF. Expression values from Affymetrix Dros2 microarrays were obtained using Plier [21], and Vampire [22] was used to detect significant expression differences at 1% FDR (See Methods and Figure S3A). RT-PCR was performed on several genes, confirming their differential expression (See Text S1 and Figure S3B). Table 1 summarizes the distribution of differentially expressed genes relative to control across the three developmental stages. (Tables listing all differentially expressed genes identified for the H and HR populations in the three developmental stages are available upon request.). There are relatively few differences in adult and larva samples, except for down-regulated genes in adult HR, which largely mapped to annotations related to peptidase activity, oxidation reduction and host defense. Among the 48 down-regulated oxidation-reduction genes in HR were seven genes related to the electron transport chain, suggesting a possible response to “perceived” hyperoxia. Differentially expressed genes shared by H and HR mapped to host defense and oxidation reduction, as well as xenobiotic metabolism; there was also a shared reduction in oogenesis-related genes in the post-eclosion stage (Table S3A). The large majority of significant expression differences were seen in post-eclosion H flies. Figure 2A summarizes the main GO-BP annotations for differentially expressed genes in post-eclosion H flies. Up-regulated genes relate primarily to development and metabolism, the latter including processes which compensate for reduced aerobic glycoysis. Down-regulated genes are highly enriched for DNA replication, cell cycle and DNA repair, contributing to the reduced size and delayed maturation observed in AF maintained at 5% O_2_ [7] and positing a reduced energetic investment in gametocyte production, as observed in a model of mitochondrial disease [23]. A more modest reduction in cell cycle genes was detected in HR flies using STEM [24] (Short Time-series Expression Miner) analysis (see Text S1 and Figure S3C), suggesting that the decreased DNA replication in AF may be at least partially genetic/epigenetic in origin. This could account for the reduction in oogenesis gene expression seen in both AF populations (Table S3A), reflecting polymorphisms detected in oogenesis and cell cycle genes (Table S1B).

**Table 1:** Summary of Gene Expression Differences by Developmental Stage.

Comparison^1^	Adult	Post-Eclosion	Larva
Number Up-regulated			
H *vs* C	339	2149	198
HR *vs* C	207	56	142
H-HR Common	117	45	83
Number Down-regulated			
H *vs* C	342	1992	233
HR *vs* C	616	75	151
H-HR Common	200	52	102

1- Data reflect genes significant using a FDR 1% cutoff, for which the mean Control expression ≥64.

### The Wnt pathway is highly represented among differentially expressed genes

Pathway analysis of the post-eclosion H set of differentially expressed genes identified several developmental/signaling pathways showing multiple gene involvement (Figure 2B, Table S3B). While no KEGG or PANTHER signaling pathway was significantly enriched, the Wnt pathway ranked second according to both pathway databases and together they identified a total of 30 up-regulated and 22 down-regulated Wnt pathway genes. GO-BP identified an additional 10 up-regulated and 6 down-regulated genes. Table S4 summarizes the expression changes observed and the Wnt pathway association for these 68 genes. We consider the identification of numerous differentially expressed Wnt pathway genes to support the genomic analysis and thereby provide additional justification to investigate experimentally whether Wnt signaling plays a role in adaptation to hypoxia.

### Genetic upregulation of Wnt pathway signaling increases *Drosophila* eclosion under hypoxia

Wnt signaling consists of at least three pathways which control a variety of developmental processes: Canonical (activates transcription via *β-catenin*/*arm* stabilization), Planar Cell Polarity (PCP), and Wnt/Ca^++^ signaling pathways [25], [26], [27]. Figure 1D highlights core Wnt pathway genes displaying polymorphisms and/or post-eclosion differential expression. Although polymorphisms and gene expression differences are seen in all three of these pathways, the canonical pathway is best described and has been implicated in several human disorders, including colorectal and other cancers [28], [29], [30]. The gene expression changes observed in the post-eclosion AF provide evidence for both activation and suppression of the canonical pathway. This is not surprising given that regulation of Wnt signaling is tissue- as well as time-dependent and the expression changes observed represent a mixture of fly tissues. We noted that the canonical pathway co-receptors *arr* and *dally* were both up-regulated and, in the presence of Wnt signaling up-regulation of the *Tcf* homolog, *pang*, which otherwise may inhibit Wnt target gene expression [30], would potentiate the pathway. We therefore genetically manipulated the canonical pathway using the Gal4-UAS system [31] to determine whether Wnt activation would affect the ability of naïve or unadapted flies to tolerate a 5% O_2_ environment. A 5% O_2_, rather than 4% O_2_, environment was selected based on our experience that the former presents a hypoxic stress severe enough to restrict eclosion of control flies but not so severe as to preclude an impact by single gene effects. After two days at 21% O_2_ for egg-laying, adults were removed and culture tubes were transferred to a controlled 5% O_2_ chamber. After 3–4 weeks, we assessed hypoxia tolerance by measuring the rate of adult eclosion, the stage in development we have found most sensitive to hypoxia. Successful eclosion is thus a key step in adaptation to chronic hypoxia, and improved eclosion rates increase the odds of producing sufficient progeny to perpetuate the adapting population. We selected for study those genes that were available as homozygous UAS strains and that represented strategic locations within the Wnt pathway; fly strain information is provided in Table S5A and experimental details in Table S5B.

**Figure 3:**
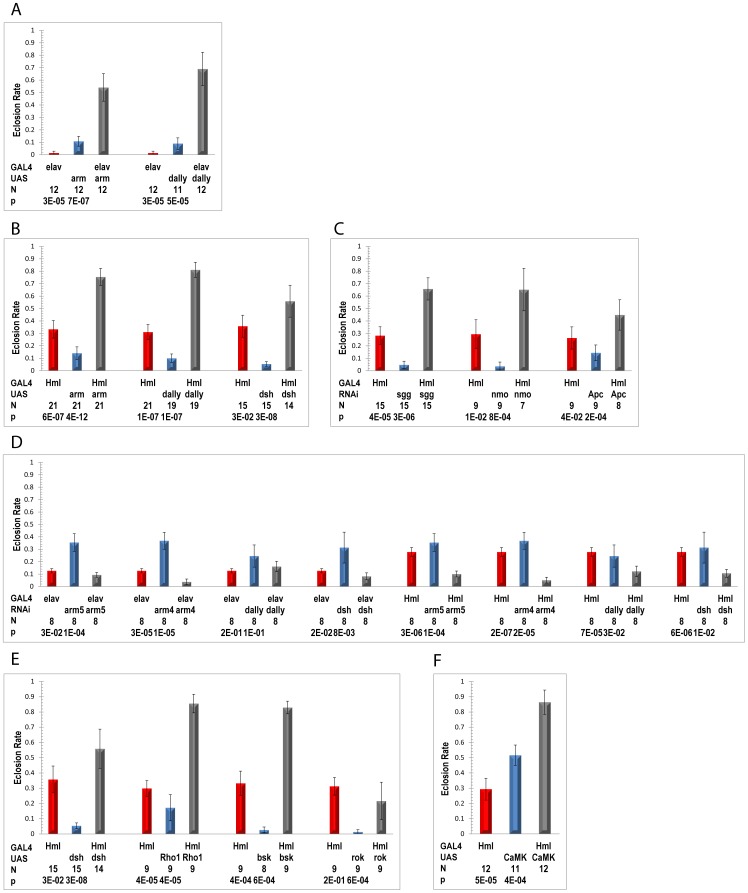
Wnt pathway activation increases adult eclosion rate of flies grown in 5% O_2_. (A–F) Number of replicates (N), each with ≥15 pupae, is indicated below each strain description. Eclosion rate for each cross is compared with that of its respective, concurrently tested parental strains using Wilcoxon test (5% experiments) or Welch t-test (6% experiments). Bars represent mean ±1.96 SEM (95% C.I) (See Methods and Table S5B for details). Eclosion rates in 21% O_2_ are generally >90%. (A) Neuron-specific overexpression of canonical pathway activators *arm* and *dally* driven by *elav*-GAL4. (B) Hemocyte-specific overexpression of canonical pathway activators *arm*, *dsh* and *dally* driven by *Hml*-GAL4. (C) Hemocyte-specific knockdown of canonical pathway inhibitors *sgg*, *nmo* and *Apc* driven by *Hml*-GAL4. (D) Neuron or hemocyte-specific knockdown of canonical pathway activators, *arm*, *dsh* and *dally* driven by *elav*-GAL4 or *Hml*-GAL4, respectively. Two different Rnai stocks, designated as arm5 and arm4, were used to test *arm*, the key mediator of the Wnt canonical pathway (See Table S5A). (E) Hemocyte-specific expression of PCP pathway activators *dsh* (also a canonical pathway activator), *Rho1*, *bsk*, and *rok* driven by *Hml*-GAL4. (F) Hemocyte-specific expression of Wnt Ca++ pathway activator *CaMKII* driven by *Hml*-GAL4.

We initially focused on altering gene expression in neurons, a target tissue for hypoxia rescue in *C elegans* [32]. Figure 3A shows the results of overexpression of canonical Wnt pathway activators *arm* and *dally*. The data show that activation of canonical Wnt signaling in neurons leads to significantly increased adult eclosion. Mean eclosion rates for parental lines were significantly lower than rates for their respective cross progenies, with p-values ≤3×10^−5^.

Hemocytes, the Drosophila counterpart of mammalian monocytes, produce several antimicrobial peptides (AMP) as well as release *Spz* to activate Toll signaling in the fat body [33], and play a key role in tissue remodeling during metamorphosis by removing apoptosing larval tissues [34]. In addition, there has been evidence for an interaction between hypoxia responsiveness and the immune system [35]. Given the shared differential expression of defense-related genes by AF populations, both those maintained in hypoxia and those reverted to normoxia, and results from our laboratory showing a role for hemocytes in severe hypoxia [36], we next asked whether Wnt activation in hemocytes might also impact hypoxia tolerance. In addition to the genes studied in neurons, we tested the effect of *dsh* overexpression, and knockdown of *sgg*, *apc* and *nmo*, using the larval hemocyte driver, *Hml*-Gal4. Figures 3B and 3C show, respectively, that canonical pathway activator overexpression and inhibitor knockdown in hemocytes also leads to significantly increased adult eclosion, with p-values of cross progenies *vs* parental strains ranging from 4×10^−2^ to 4×10^−12^. Conversely, we show that knockdown of pathway activators *arm, dsh and dally* in hemocytes, and of *arm* and *dsh* in neurons, leads to a significant reduction in eclosion rate in 6% O_2_ relative to both parental strains (Figure 3D; p-values ranging from 3×10^−2^ to 2×10^−7^).

Using the *Hml*-Gal4 driver, we also studied the effect of overexpression of several PCP pathway genes and a Wnt Ca^2+^ pathway gene. As shown in Figure 3E, overexpression of *Rho1* and *bsk* (Jnk), in addition to *dsh*, but not *rok*, were associated with significantly increased adult eclosion, with p-values ranging from 3×10^−2^ to 3×10^−8^. In addition, overexpression of *CaMKII* (Figure 3F) increased adult eclosion in 5% O_2_ (p≤4×10^−4^). Figure 1D indicates the location within the Wnt pathways of the genes studied. Although several Wnt pathway genes participate directly or via cross-talk with other signaling pathways, the fact that eclosion rate is increased by activation of these pathways at multiple sites provides strong support that activation of Wnt signaling per se increases hypoxia tolerance.

In contrast with the effect of Wnt signaling on hypoxia tolerance during development, and in agreement with an absence of Wnt pathway gene overexpression in the hypoxia-tolerant adults, we did not find evidence that overexpression of *arm* in hemocytes or neurons increases adult short-term tolerance of anoxia or hypoxia. In particular, F1's did not recover from anoxic stupor faster than both parental lines (see Text S1 and Figure S4), and did not demonstrate consistently better activity in 2% O_2_ (see Text S1 and Supplementary Movies S1 and S2). A similar disparity between eclosion rate and recovery from anoxia was observed among the 27 isogenic parental lines used in selection of the hypoxia-tolerant flies (Figures S1 and S2 in reference 6).

## Discussion

Recent work describes at least two points of intersection between hypoxia and Wnt signaling. In colon carcinoma lines, hypoxia (1% O_2_) causes *HIF-1α*, the major regulator of the transcriptional response to hypoxia [37], [38], [39], to compete with *TCF-4* for binding to *β-catenin*; this causes an increase in *HIF-1α*-mediated transcription while decreasing *TCF-4* mediated transcription [40], which the authors suggest promote survival and adaptation to hypoxia and contribute to the cell cycle arrest induced by hypoxia, respectively. In contrast to its effect on differentiated cells, hypoxia, acting via *HIF-1α*, has been reported to enhance canonical Wnt signaling in embryonic stem cells and neural stem cells, promoting stem cell proliferation and Wnt-regulated differentiation [41], [42]. In this work, we demonstrate a new mechanism of hypoxia adaptation, involving Wnt pathway activation, which promotes hypoxia tolerance during fly development.

Although normal adult flies are relatively tolerant to hypoxia and able to survive in 5-6% O_2_, we have observed that two stages in the developmental cycle are particularly vulnerable to hypoxia – hatching of the embryo into the first instar larva, and adult eclosion from pupae. Acquisition of the ability to transit these stages under reduced O_2_ requires changes in signaling and gene expression that precede these developmental landmarks, and enable the developing fly to match the energy requirements of eclosion with the energy available to it in its hypoxic environment. An improvement in the rate of adult eclosion in 5% O_2_ was detected upon selectively activating Wnt pathway signaling in two different cell types, neurons and hemocytes. Conversely, knockdown of Wnt pathway activators reduced the eclosion rate in 6% O_2_ in these two cell types.

Given the vulnerability of brain tissue to an inadequate oxygen supply, neurons are a classic target for studies of hypoxia rescue [32]. Our results demonstrate that neuron-specific overexpression of Wnt canonical pathway activators leads to a significant increase in adult eclosion rates. It is possible that alterations in the number and type of neurons produced as a result of hypoxia-stimulated Wnt signaling [42] may contribute to the observed increased ability to survive in a hypoxic environment. Regardless, our ability to increase hypoxia tolerance by activating canonical Wnt signaling exclusively in neurons raises the possibility of targeting this pathway in solid brain tumors where cells differentiate and proliferate under severely hypoxic conditions.

We have also shown that activation of the Wnt canonical or PCP pathway in hemocytes promotes increased adult eclosion in 5% O_2_. Although host defense and innate immunity have been linked to hypoxia [35], it is possible, given their critical role in morphogenesis [34], that hemocyte physiology is specifically targeted. In this regard, canonical Wnt signaling has been shown to maintain hemocyte precursors both directly by preventing their differentiation, and indirectly by promoting proliferation and maintenance of cells in the hematopoietic microenvironment that maintain precursor stemness [43]; more prolonged precursor status would allow for increased rounds of replication and eventual increased hemocyte numbers. Noncanonical Wnt signaling also impacts the hemocyte population, as *Rac1* overexpression, acting via *bsk,* was shown to increase the number of circulating hemocytes by mobilizing the sessile hemocyte population [44]; Rac1 also increases canonical Wnt signaling by promoting *arm* nuclear translocation, either directly or by activated *bsk* phosphorylation of *arm* [45]. Finally, *Rac1* and *Rho1* GTPases, which both help regulate shape and migration of Drosophila hemocytes [46], can reciprocally induce each other: *Rac1* acting via *bsk*, and *Rho1* acting via *dia* [47].

In our earlier study, we identified Notch signaling as important to the development of hypoxia-tolerance [5]. Numerous studies have noted that Notch-canonical Wnt cross-talk may be antagonistic or cooperative, depending on the cellular/developmental context [48], [49], [50], [51], [52], [53], [54], [55]. Given the diversity and ubiquity of interactions between Notch and Wnt in development and disease, it has been proposed that Wnt and Notch signaling comprise an integrated cellular system (Wntch) to exert mutual control in the determination of cell fate [54]. Depending on the tissues and developmental stages during which changes in Notch and/or canonical Wnt signaling act to promote hypoxia tolerance, either pathway might be activated or repressed, with the two pathways cooperating or antagonistic.

An extensive literature documents the central role HIF-1 plays in fostering hypoxia tolerance in adult organisms [37], [38], [39], and more recently positive allele selection of several HIF-1 pathway genes has been identified in Tibetans adapted to high-altitude residence [56], [57]. Here we highlight the importance of changes during development for adaptation to chronic hypoxia by integrating an analysis of genomic changes identified in flies adapted to 4% O_2_ with a transcriptomic analysis at three developmental stages. The observation, that Wnt pathway proteins figure prominently among both post-eclosion differentially expressed genes and genes with ≥1 polymorphisms, led to our hypothesis concerning its role in hypoxia tolerance. We validated our hypothesis through genetic cross experiments, demonstrating that Wnt signaling plays a significant role in evolution towards a hypoxia tolerant phenotype in flies. Several studies suggest that our results are relevant for tumor adaptation to hypoxia. It has long been known that Wnt pathway-activating mutations occur in numerous tumor types, most notably colorectal cancer [58]. More recently, interactions between HIF-1 and β-catenin identified in a variety of cancers under hypoxic conditions have been found to promote tumor cell protection, progression, and metastatic potential [59], [60], [61], amplifying the interest in Wnt signaling as a therapeutic target for cancer [62], [63], [64].

## Materials and Methods

### Drosophila stocks and cultures

*Elav* and *Hml* GAL4 driver lines and UAS-expressor lines (*arm*, *dsh*, *dally*, *Rho1*, *bsk*, *rok*, *CaMKII*) were obtained from Bloomington Drosophila Stock Center (http://flystocks.bio.indiana.edu/). UAS-RNAi lines were obtained from the Vienna Stock Collection (http://stockcenter.vdrc.at/control/main; *sgg*, *nmo*) or Bloomington (*apc*, *arm*, *dsh*, *dally*). Stocks were cultured on standard media.

### Whole-genome resequencing

Genomic DNA was isolated from pools of 100 male and 100 female adult-flies collected from AF or generation-matched control populations, as described in Zhou et al [5]. Paired-end sequencing was performed using the Illumina Genome Analyzer II and sequencing kit v3. The genome resequencing data is available at NCBI's Sequence Read Archive (SRA), accession number SRP004819 (http://www.ncbi.nlm.nih.gov/sra?term=SRP004819).

### Genomic sequencing data analysis

The next-generation sequencing data for each of the pools was derived from 200 flies descended from 27 parental strains. Maq v.0.7.1 [9] was used under its default parameters to map reads from two control (C1, C2) and two hypoxia-tolerant (H1, H2) populations to the *Drosophila melanogaster* reference genome release 5.16 downloaded from FlyBase release 5.16 [10]. Since neither C1 nor C2 was under selective pressure, these two populations would be expected to differ only as the result of genetic drift. The C2 data was found to have much lower high-quality coverage (as defined below) and was not used in subsequent analyses.

Data from paired-end sequencing was filtered to remove duplicate reads. SNP calling was limited to “high-quality” loci with ≥10X coverage, Maq-reported base quality ≥20, and Maq-reported best read quality ≥40 for all three populations: C1, H1 and H2 (“evaluable loci”). Approximately 50% of euchromatin loci met these conditions. A SNP was called if the following criteria were met: 1) H1 and H2 base differed from both C1 and reference; and 2) the H1 (H2) base was identified in ≥90% of H1 (H2) reads and in ≤10% of the C1 reads. Although not specifically required, for all SNPs called the H1 and H2 bases were identical. A FDR was estimated for the evaluable loci by identifying loci meeting these criteria: 1) H1 (H2) base differed from reference; 2) the H1 (H2) base was identified in ≥90% of H1 (H2) reads and in ≤10% of the H2 (H1) reads. Consistent indels were generated and corrected for homopolymer tracts using Maq software for each of the populations, C1, H1 and H2. Software was written to select indels meeting the following criteria: 1) indel is Maq-determined type “*” (confirmed by reads from both strands) or “+” (≥2 reads from same strand); 2) locus is covered by ≥10 reads in both H1 and H2 datasets; 3) ≥90% of H1 and H2 reads have the indel; 4) indel is not identified in C1. Identified SNPs and indel loci were mapped to genes/gene regions using fasta files downloaded from FlyBase [10]. Coding region SNPs were evaluated using the Ensembl *D. melanogaster* BDGP5.13.56.gtf file [65].

### Affymetrix microarray analysis

Twenty-seven samples from hypoxia-selected flies maintained in 4% O_2_ (H) or reverted for five generations to normoxia (HR) and control flies (C) were used for microarray experiments. In each case, three replicate pools of flies were collected from three developmental stages: larva (L: 25/pool); 0.5–3 hours post-eclosion (Ec: 25 females and 25 males/pool); and 7–9 day adults (A: 25 females and 25 males/pool). Total RNA was extracted using TRIzol (Invitrogen, Carlsbad, CA) followed by a clean-up with RNeasy kit (Qiagen, Valencia, CA). Total RNA was adjusted to 5 µg/15 µl prior to analysis. Affymetrix Drosophila Genome 2.0 arrays were used and probe labeling, array hybridization, and image scanning were performed following the standard protocol according to manufacturer's instruction (Affymetrix, Santa Clara, CA). Gene expression values (probeset summary values) were generated using the Plier algorithm [21] implemented in the Affymetrix Expression Console software (Affymetrix, Santa Clara, CA) and differential expression between experimental groups (C vs H, C vs HR and HR vs H) was determined using VAMPIRE software [22]. For each comparison, VAMPIRE-reported significant probesets with a mean baseline (control) expression <64 were excluded from further analysis due to poor correlation among biological replicate samples (Figure S3A). The microarray data is available at NCBI's Gene Expression Omnibus (GEO), accession number GSE36507 (http://www.ncbi.nlm.nih.gov/geo/).

### Functional annotation

The Database for Annotation, Visualization and Integrated Discovery (DAVID) [11], [12] software was used for functional annotation of genetic polymorphisms and differentially expressed genes. Flybase gene IDs (polymorphisms) or Affymetrix probe IDs (microarray) were submitted and evaluated for enrichment against the *D. melanogaster* background. KEGG Wnt-pathway enrichment was assessed by calculating the hypergeometric p-value for polymorphism enrichment (p = 4.56e–06), asking whether the number of polymorphic loci in KEGG Wnt pathway genes was significant, given the total number of polymorphic loci in KEGG pathway genes, the total number of loci in KEGG Wnt pathway extended genes, and the total number of KEGG pathway extended gene loci.

### Hypoxia tolerance testing

#### Eclosion Rate

The impact of Wnt-pathway activity on survival in hypoxia was assessed by the ability of flies to eclose after culture in a hypoxic environment. Since classical loss-of-function or gain-of function mutations produce a global effect, which is often lethal when dealing with genes critical to development, we opted to use the UAS-GAL4 system to selectively over- or underexpress genes in specific cell types. UAS(-RNAi) stocks were crossed with elav or Hml GAL4 driver lines to produce F1 flies that express the UAS insert in neurons or larval hemocytes, respectively. The effect of Wnt-pathway activation on hypoxia survival was studied at 5%-O2. Each cross contained 5–10 virgin females and 5–10 males. Except for UAS-arm and UAS-dally (with inserts on the X-chromosome), GAL4 virgin females and UAS males were used. UAS-arm and UAS-dally virgin females were crossed with males containing the elav-Gal4 insert on the 3rd chromosome (driver 8760).Flies were allowed to lay eggs for approximately 48 hours in normoxia; adults were then transferred to fresh vials and the vials containing eggs were moved to a room temperature chamber, computer-controlled to maintain a 5% oxygen atmosphere. Three replicate vials were set up for each cross and adults from each cross laid one or more clutches of eggs over 48-hour periods. Parental lines for each cross were tested in parallel as controls. After 3–4 weeks, vials from both crosses and parental lines were evaluated for eclosion rate. Experiments were excluded if a parental (control) rate of eclosion was >0.70. Only vials with ≥15 total pupae were included. Numbers of eclosed (empty pupal case) and non-eclosed pupae were counted and the ratio between eclosed and total number of pupae was calculated. Each data point presented reflects averaged data from 5–23 replicate tubes, each with ≥15 pupae, derived from 3–7 separate experiments reflecting at least two independent matings. Since some of the datasets were not normally distributed (Shapiro test), statistical significance was calculated using the non-parametric Wilcoxon Rank Sum Test (R Statistical Package [66]); the Wilcoxon Test with continuity correction was used with datasets containing ties for which exact p-values could not be calculated. Experiments looking at the effect of Wnt-pathway inhibition on hypoxia survival followed a similar protocol, but were conducted at 6%-O2 to ensure a sufficiently high parental background eclosion rate. Two different Rnai stocks (Bloomington stock numbers 31305 and 35004) were used to test *arm*, the key mediator of the Wnt canonical pathway; they are designated as arm5 and arm4 in Figure 3D. Each data point reflects averaged data from 8 replicate tubes derived from a single experiment. Since data were normally distributed, statistical significance was calculated using the two-sided Welch (unequal variance) t-test. In all cases, data are presented as the mean of all replicates ±1.96 SEM (95% C.I.) [67]. Strain information for flies used in these experiments is provided in Table S5A; number of tubes, total number of pupae, and statistical details for each genetic cross experiment are provided in Table S5B.

## Supporting Information

Figure S1 **Expanded network of Notch interactors.** Interrogation of the Costello high confidence 20K network using the original resequencing analysis revealed a connected subnetwork of 287 polymorphism-containing genes, of which 25 (colored red) directly interact with Notch. Inclusion of non-polymorphic genes highly connected (≥5 interactions) to genes containing polymorphisms identified an additional 49 Notch interactors (colored blue). Annotation revealed that this set of genes included subsets that participate in several signaling pathways in addition to the Notch pathway.
(PDF)

Figure S2 **Overview of SNP/indel analysis procedure.** Approximately 50% of the euchromatin portion of the five major gene-bearing chromosomes (X, 2L, 2R, 3L, 3R; range 45.4–53.4%) met the coverage and quality criteria in the C dataset as well as in both H1 and H2 datasets and were analyzed for SNPs and small indels.
(PDF)

Figure S3 **Analysis of Expression Data.** *Figure S3A* shows the correlation between two log2-transformed control biological replicate datasets. (A1) All data and (A2) data with expression values > = 64. Elimination of expression values <64 greatly improves the correlation between sets, increasing confidence in the results. Therefore, VAMPIRE-reported significant probesets with a mean baseline (control) expression <64 were excluded from further analysis. *Figure S3B* provides PCR Confirmation of Post-Eclosion Differential Expression in Hypoxia-Adapted Flies. Three biological replicates of each condition (C, H and HR) were tested in triplicate for the indicated genes using actin to normalize expression values. Data is presented as (B1) fold change of H or HR over C and (B2) mean relative expression ± SD. All tested genes were significant by microarray for post-eclosion H flies; only CG13422 was significant by microarray for post-eclosion HR. Significance in the PCR assay (p≤0.05) was determined by two-tailed t-test; all tested genes for post-eclosion H flies were significant and none were significant for HR flies. Actin5C was used in all experiments except for pim, where Act88F was used. *Figure S3C* summarizes the STEM analysis of gene expression in post-eclosion flies. (C1) Each panel is a comparison of STEM-generated microarray time-series profiles identified for two conditions, original and comparator, here using a third condition as the denominator to generate ratio data. In each case, the profiles identified for the first condition (original) appear in the left-hand column. Profiles for the second condition (comparator) which contain genes appearing in an original profile are positioned in the same row, to the right of the original profile. (1) H (original) vs C (comparator); (2) H (original) vs HR (comparator); (3) HR (original) vs C (comparator). (C2) Summary of results from STEM analysis. Processes dependent on a hypoxic environment are italicized; processes that may have a genetic/epigenetic component are bolded. For Cell Cycle genes: H- 41 Probesets, 40 with fold change 0.08–0.60 and p-value <10^−4^; HR - 28 Probesets, 11 with fold change 0.65–0.77 and p-value 0.0014–0.033.
(PDF)

Figure S4 **Recovery from Anoxia.** Recovery time after 5 minutes of anoxia for each cross is compared with that of its respective, concurrently tested parental strains using two-tailed unpaired Student's t-test. (See Text S1). Bars represent the mean ±1.96 SEM (95% C.I). In neither cross did the F1 recover faster than both parental lines.
(PDF)

Table S1 **Polymorphisms Distinguishing AF from Control flies.** Table S1A: Fixed SNPs and Indels Distinguishing AF from Control Flies. Table S1B: GO-BP Annotation of Polymorphisms. Table S1C: Pathway Enrichment of AF Polymorphisms.
(PDF)

Table S2 **Wnt pathway polymorphisms.** Table S2A: SNPs in Wnt-Pathway Associated Genes. Table S2B: Indels in Wnt-Pathway Associated Genes. Table S2C: Coding region Polymorphisms in Wnt pathway-Associated Genes. Table S2D: Polymorphism-Containing Wnt Pathway-Associated Genes.
(PDF)

Table S3 **Differential Gene Expression.** Table S3A: KEGG and GO BP annotations for DE genes shared by H and HR. Table S3B: Pathway Analysis of Differential Expression in Post-Eclosion Hypoxia-Tolerant Flies Maintained in 4% O2.
(PDF)

Table S4 **Wnt Pathway-Associated Genes Differentially Expressed in Post-Eclosion Hypoxia Tolerant Flies Grown at 4% O2.**
(PDF)

Table S5 **Details of genetic experiments.** Table S5A: Fly strains used in genetic cross experiments. Table S5B: Eclosion Rate Experimental Details.
(PDF)

Text S1 **Supplementary Methods.**
(DOCX)

Movie S1 **UAS-arm X Hml-Gal4 cross.** Groups of 7–10 adult males (5–6 days old) were acclimated to 2% O_2_ and their activity recorded (See Text S1). The tubes from left to right contain UAS-arm, Hml-Gal4 and F1 flies. The F1 does not show consistently better activity than both parental lines.
(MOV)

Movie S2 **UAS-arm X Elav-Gal4 cross.** Groups of 10 adult males (8–9 days old) were acclimated to 2% O_2_ and their activity recorded (See Text S1). The tubes from left to right contain UAS-arm, elav-Gal4 and F1 flies. The F1 does not show consistently better activity than both parental lines.
(MOV)
