# Supplementary material for: Wnt Pathway Activation Increases Hypoxia Tolerance during Development
Source: PLoS One. 2014 Aug 5;9(8):e103292. doi: 10.1371/journal.pone.0103292 (PMC4122365; doi:10.1371/journal.pone.0103292)
Supplement: Figure S1 — Expanded network of Notch interactors. Interrogation of the Costello high confidence 20K network using the original resequencing analysis revealed a connected subnetwork of 287 polymorphism-containing genes, of which 25 (colored red) directly interact with Notch. Inclusion of non-polymorphic genes highly connected (≥5 interactions) to genes containing polymorphisms identified an additional 49 Notch interactors (colored blue). Annotation revealed that this set of genes included subsets that participate in several signaling pathways in addition to the Notch pathway. (PDF) [file pone.0103292.s001.pdf]

Figure S1

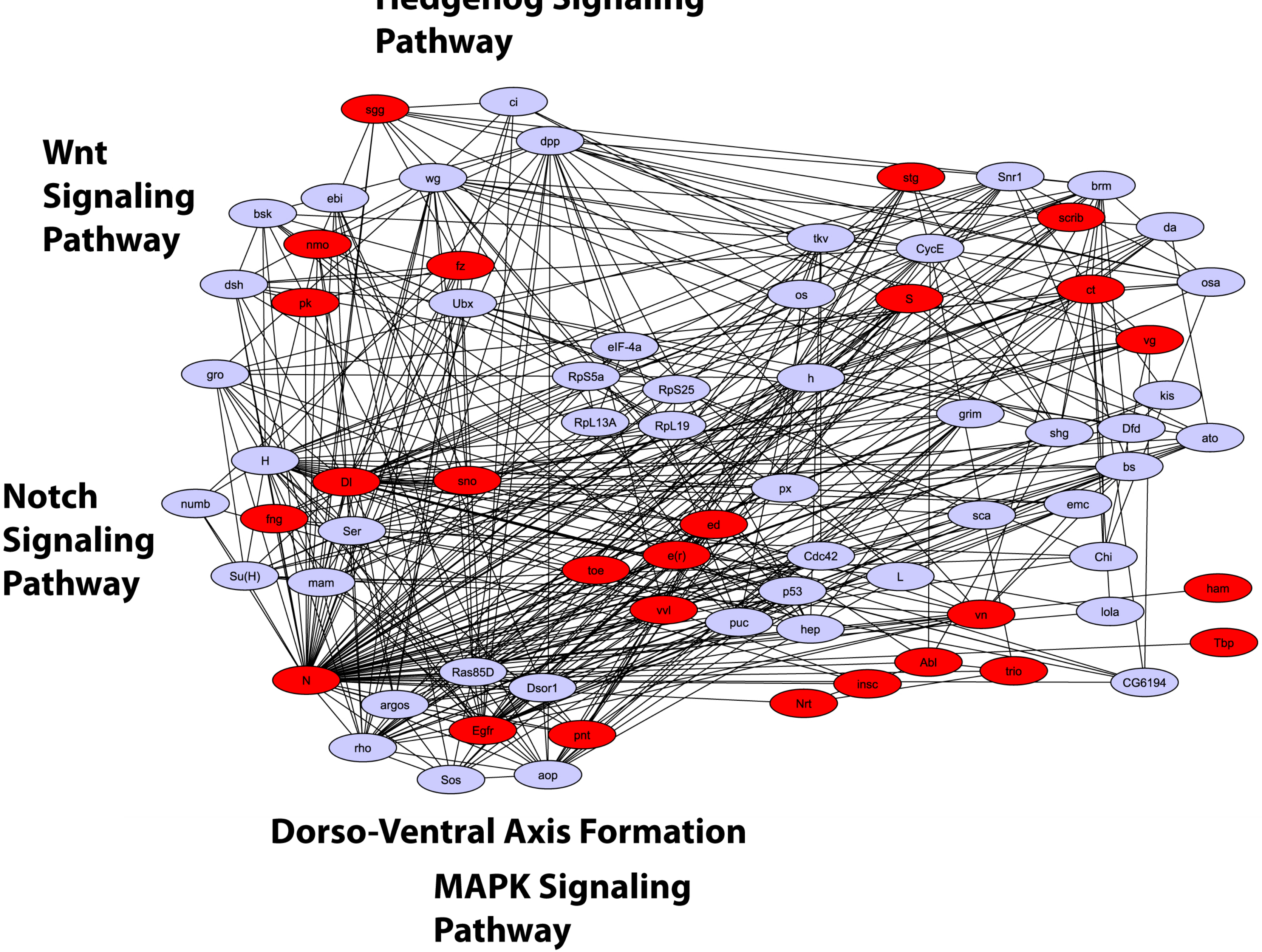

Hedgehog Signaling Pathway
Wnt Signaling Pathway
Notch Signaling Pathway
Dorso-Ventral Axis Formation
MAPK Signaling Pathway
sgg
ci
dpp
wg
ebi
bsk
nmo
fz
dsh
pk
Ubx
stg
Snr1
brm
scrib
da
tkv
CycE
os
S
ct
osa
eIF-4a
vg
gro
RpS5a
RpS25
h
kis
RpL13A
RpL19
grim
shg
Dfd
ato
H
Dl
sno
bs
numb
px
fng
Ser
ed
sca
emc
e(r)
Cdc42
Chi
toe
Su(H)
mam
L
p53
ham
vvl
puc
hep
vn
lola
Tbp
Abl
trio
Ras85D
N
insc
CG6194
Dsor1
Nrt
argos
Egfr
pnt
rho
Sos
aop
