# Supplementary figures and images for: Wnt Pathway Activation Increases Hypoxia Tolerance during Development
Source: PLoS One. 2014 Aug 5;9(8):e103292. doi: 10.1371/journal.pone.0103292 (PMC4122365; doi:10.1371/journal.pone.0103292)

Figure S2

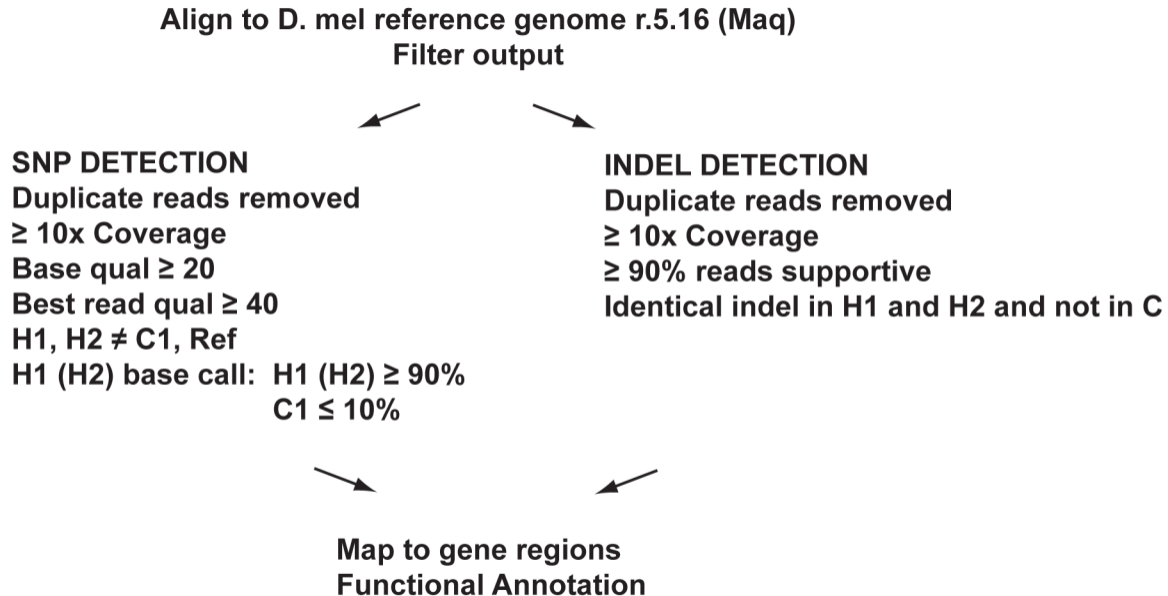

Supplement: Figure S2 — Overview of SNP/indel analysis procedure. Approximately 50% of the euchromatin portion of the five major gene-bearing chromosomes (X, 2L, 2R, 3L, 3R; range 45.4–53.4%) met the coverage and quality criteria in the C dataset as well as in both H1 and H2 datasets and were analyzed for SNPs and small indels. (PDF) [file pone.0103292.s002.pdf]

Figure S4

## Recovery From Anoxia

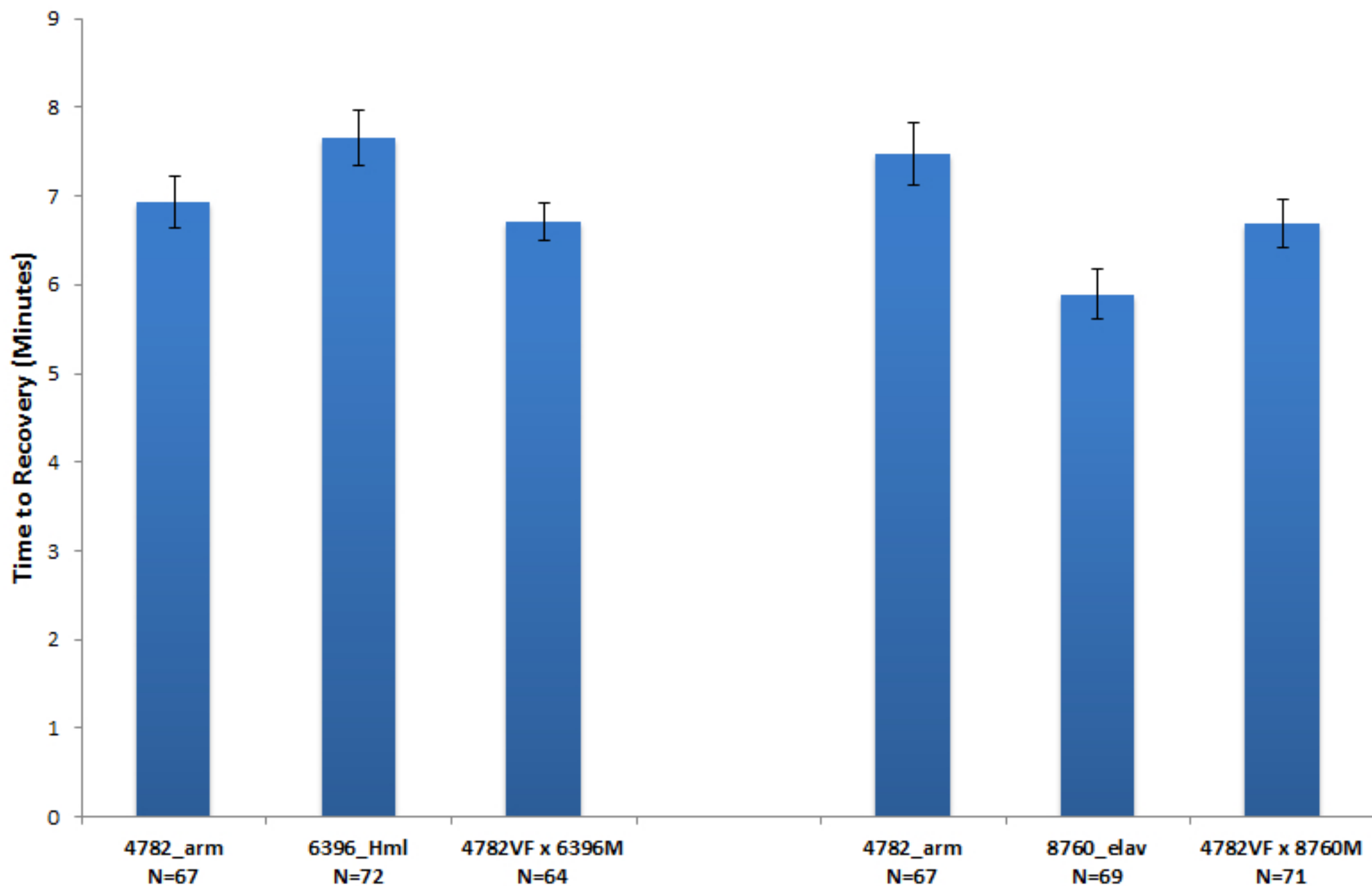

Supplement: Figure S4 — Recovery from Anoxia. Recovery time after 5 minutes of anoxia for each cross is compared with that of its respective, concurrently tested parental strains using two-tailed unpaired Student's t-test. (See Text S1). Bars represent the mean ±1.96 SEM (95% C.I). In neither cross did the F1 recover faster than both parental lines. (PDF) [file pone.0103292.s004.pdf]
