# Supplementary material for: Wnt Pathway Activation Increases Hypoxia Tolerance during Development
Source: PLoS One. 2014 Aug 5;9(8):e103292. doi: 10.1371/journal.pone.0103292 (PMC4122365; doi:10.1371/journal.pone.0103292)
Supplement: Figure S3 — Analysis of Expression Data. Figure S3A shows the correlation between two log2-transformed control biological replicate datasets. (A1) All data and (A2) data with expression values > = 64. Elimination of expression values <64 greatly improves the correlation between sets, increasing confidence in the results. Therefore, VAMPIRE-reported significant probesets with a mean baseline (control) expression <64 were excluded from further analysis. Figure S3B provides PCR Confirmation of Post-Eclosion Differential Expression in Hypoxia-Adapted Flies. Three biological replicates of each condition (C, H and HR) were tested in triplicate for the indicated genes using actin to normalize expression values. Data is presented as (B1) fold change of H or HR over C and (B2) mean relative expression ± SD. All tested genes were significant by microarray for post-eclosion H flies; only CG13422 was significant by microarray for post-eclosion HR. Significance in the PCR assay (p≤0.05) was determined by two-tailed t-test; all tested genes for post-eclosion H flies were significant and none were significant for HR flies. Actin5C was used in all experiments except for pim, where Act88F was used. Figure S3C summarizes the STEM analysis of gene expression in post-eclosion flies. (C1) Each panel is a comparison of STEM-generated microarray time-series profiles identified for two conditions, original and comparator, here using a third condition as the denominator to generate ratio data. In each case, the profiles identified for the first condition (original) appear in the left-hand column. Profiles for the second condition (comparator) which contain genes appearing in an original profile are positioned in the same row, to the right of the original profile. (1) H (original) vs C (comparator); (2) H (original) vs HR (comparator); (3) HR (original) vs C (comparator). (C2) Summary of results from STEM analysis. Processes dependent on a hypoxic environment are italicized; processes that may have a ge [file pone.0103292.s003.pdf]

Figure S3

A1

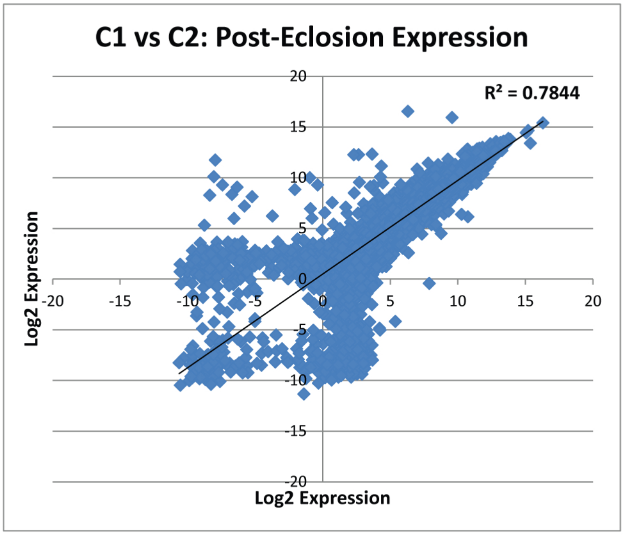

A2

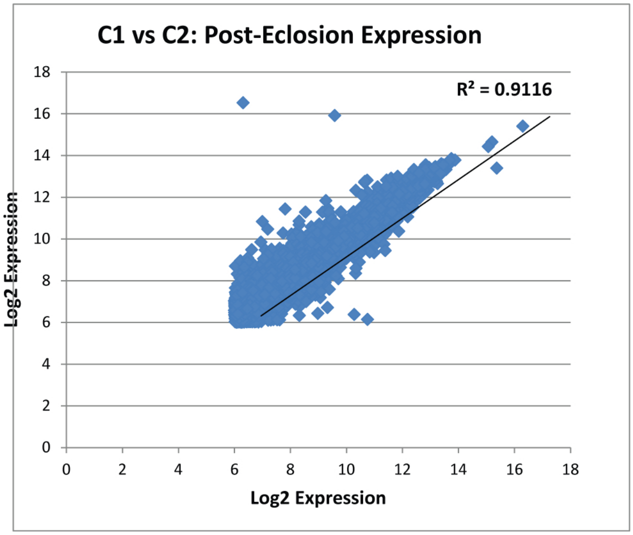

B1

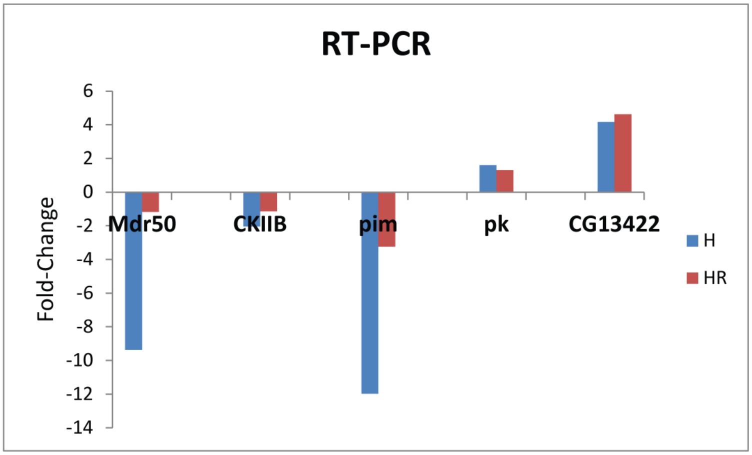

B2

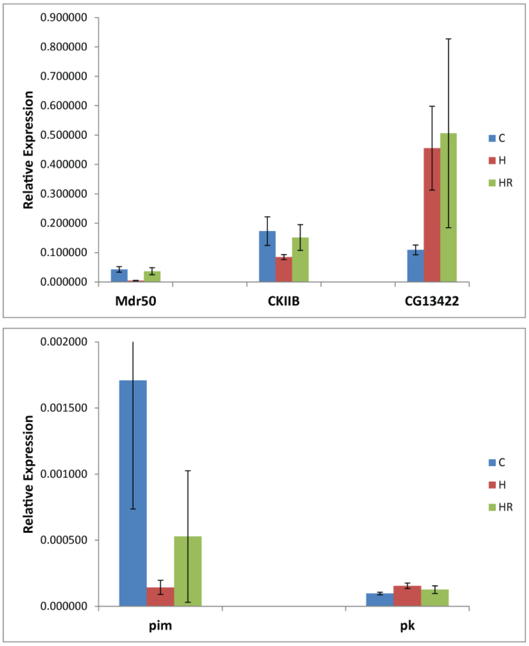

C1

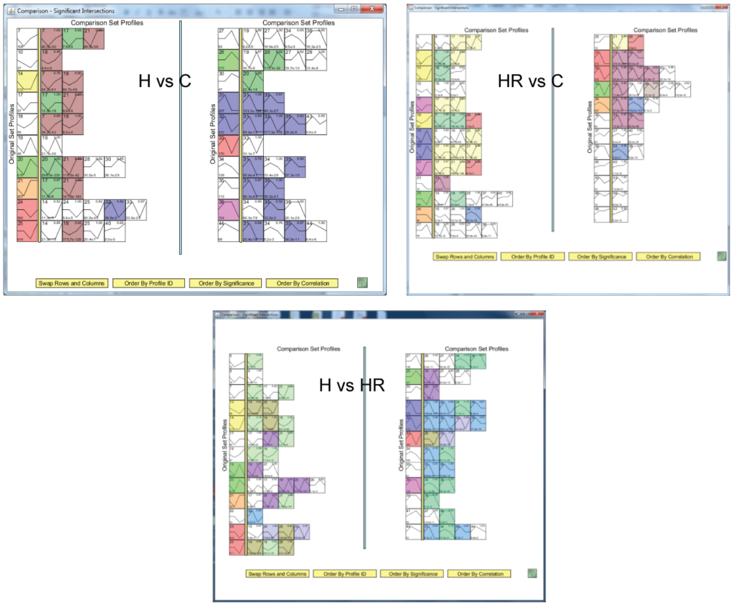

C2

	H *vs* HR (Control=C)	HR *vs* C (Control=H)	H *vs* C (Control=HR)
O_2 Exposure Difference	*YES*	No	*YES*
Genetic Difference	No	**YES**	**YES**
Ribosome biogenesis	*Decreased in H*		*Decreased in H*
Development (various)	*Increased in H*		*Increased in H*
DNA replication		**Decreased in HR***	**Decreased in H**
Cell cycle		**Decreased in HR***	**Decreased in H**
EtOH, hexose metab; oxidoreductase		Increased in HR	

* Not detected by Vampire using FDR-1% significance
