# Supplementary material for: Wnt Pathway Activation Increases Hypoxia Tolerance during Development
Source: PLoS One. 2014 Aug 5;9(8):e103292. doi: 10.1371/journal.pone.0103292 (PMC4122365; doi:10.1371/journal.pone.0103292)
Supplement: Table S1 — Polymorphisms Distinguishing AF from Control flies. Table S1A: Fixed SNPs and Indels Distinguishing AF from Control Flies. Table S1B: GO-BP Annotation of Polymorphisms. Table S1C: Pathway Enrichment of AF Polymorphisms. (PDF) [file pone.0103292.s005.pdf]

Table S1A: Fixed SNPs and Indels Distinguishing AF from Control Flies

Category	SNP	Indel
Total number of loci	2514	405
Loci mapped to genes: No. of loci (No. unique FBgn)		
Extended gene	1940 (921)	283 (320)
Upstream (2000 bp)	507 (315)	67 (71)
Downstream (2000 bp)	471 (291)	57 (58)
5' UTR	80 (55)	15 (14)
3' UTR	63 (48)	13 (16)
Intron	971 (337)	216 (185)
Exon	743 (333)	29 (29)
CDS	625 (288)	3 (3)
NS change	135 (99)	2 (2)

Abbreviations used in table: *SNP* - Single Nucleotide Polymorphism (in the context of this paper, SNP refers to loci where both adapted flies differ from both the D. melanogaster Reference and Control genomes); *FBgn* - FlyBase gene identifier (number); *Extended gene* - Flybase gene + 2000 bases upstream and downstream; *UTR* - Untranslated Region; *CDS* - Coding sequence; *NS* - Non-synonymous (change)

Table S1B: GO-BP Annotation of Polymorphisms.

Category	Term	Count	PValue	Fold Enrichment	Benjamini	FDR
GOBP_FAT	GO:0022610~biological adhesion	41	9.95E-10	2.899	1.91E-06	1.70E-06
GOBP_FAT	GO:0030182~neuron differentiation	66	3.19E-09	2.145	3.06E-06	5.46E-06
GOBP_FAT	GO:0048666~neuron development	58	9.53E-09	2.222	6.10E-06	1.63E-05
GOBP_FAT	GO:0000904~cell morphogenesis involved in differentiation	52	2.64E-08	2.282	1.27E-05	4.52E-05
GOBP_FAT	GO:0007155~cell adhesion	36	5.17E-08	2.751	1.98E-05	8.84E-05
GOBP_FAT	GO:0030030~cell projection organization	58	6.08E-08	2.113	1.95E-05	1.04E-04
GOBP_FAT	GO:0048667~cell morphogenesis involved in neuron differentiation	49	9.48E-08	2.262	2.60E-05	1.62E-04
GOBP_FAT	GO:0007552~metamorphosis	57	1.12E-07	2.093	2.69E-05	1.92E-04
GOBP_FAT	GO:0048707~instar larval or pupal morphogenesis	55	1.55E-07	2.107	3.31E-05	2.66E-04
GOBP_FAT	GO:0000902~cell morphogenesis	65	1.65E-07	1.955	3.16E-05	2.82E-04
GOBP_FAT	GO:0048812~neuron projection morphogenesis	48	2.00E-07	2.231	3.50E-05	3.43E-04
GOBP_FAT	GO:0031175~neuron projection development	48	2.23E-07	2.224	3.57E-05	3.82E-04
GOBP_FAT	GO:0048569~post-embryonic organ development	49	2.49E-07	2.193	3.67E-05	4.25E-04
GOBP_FAT	GO:0009886~post-embryonic morphogenesis	55	2.74E-07	2.071	3.76E-05	4.69E-04

GOBP_FAT	GO:0009791~post-embryonic development	63	4.22E-07	1.930	5.41E-05	7.23E-04
GOBP_FAT	GO:0007409~axonogenesis	37	4.72E-07	2.484	5.67E-05	8.08E-04
GOBP_FAT	GO:0035120~post-embryonic appendage morphogenesis	40	9.01E-07	2.322	1.02E-04	0.0015
GOBP_FAT	GO:0048858~cell projection morphogenesis	50	9.81E-07	2.077	1.05E-04	0.0017
GOBP_FAT	GO:0035114~imaginal disc-derived appendage morphogenesis	41	1.04E-06	2.281	1.05E-04	0.0018
GOBP_FAT	GO:0048563~post-embryonic organ morphogenesis	46	1.06E-06	2.153	1.01E-04	0.0018
GOBP_FAT	GO:0007560~imaginal disc morphogenesis	46	1.06E-06	2.153	1.01E-04	0.0018
GOBP_FAT	GO:0002165~instar larval or pupal development	60	1.23E-06	1.908	1.13E-04	0.0021
GOBP_FAT	GO:0035107~appendage morphogenesis	41	1.44E-06	2.252	1.25E-04	0.0025
GOBP_FAT	GO:0048737~imaginal disc-derived appendage development	41	1.60E-06	2.243	1.34E-04	0.0027
GOBP_FAT	GO:0048736~appendage development	41	2.20E-06	2.216	1.76E-04	0.0038
GOBP_FAT	GO:0032990~cell part morphogenesis	50	2.66E-06	2.008	2.04E-04	0.0046
GOBP_FAT	GO:0007476~imaginal disc-derived wing morphogenesis	37	4.97E-06	2.256	3.67E-04	0.0085
GOBP_FAT	GO:0007472~wing disc morphogenesis	37	6.16E-06	2.236	4.38E-04	0.0105
GOBP_FAT	GO:0007411~axon guidance	27	7.34E-06	2.639	5.03E-04	0.0126

GOBP_FAT	GO:0007444~imaginal disc development	57	7.58E-06	1.839	5.01E-04	0.0130
GOBP_FAT	GO:0032989~cellular component morphogenesis	66	1.97E-05	1.694	0.0013	0.0337
GOBP_FAT	GO:0016337~cell-cell adhesion	16	2.03E-05	3.605	0.0013	0.0348
GOBP_FAT	GO:0035220~wing disc development	42	2.53E-05	1.987	0.0015	0.0433
**GOBP_FAT**	**GO:0007166~cell surface receptor linked signal transduction**	**76**	**2.60E-05**	**1.609**	**0.0015**	**0.0445**
GOBP_FAT	GO:0035239~tube morphogenesis	19	3.88E-05	3.007	0.0022	0.0664
**GOBP_FAT**	**GO:0006793~phosphorus metabolic process**	**66**	**1.34E-04**	**1.592**	**0.0073**	**0.2286**
**GOBP_FAT**	**GO:0006796~phosphate metabolic process**	**66**	**1.34E-04**	**1.592**	**0.0073**	**0.2286**
**GOBP_FAT**	**GO:0007186~G-protein coupled receptor protein signaling pathway**	**43**	**1.55E-04**	**1.821**	**0.0083**	**0.2656**
GOBP_FAT	GO:0006928~cell motion	42	1.67E-04	1.831	0.0086	0.2847
GOBP_FAT	GO:0008038~neuron recognition	13	2.01E-04	3.527	0.0101	0.3433
GOBP_FAT	GO:0008037~cell recognition	13	2.47E-04	3.457	0.0121	0.4213
GOBP_FAT	GO:0035218~leg disc development	13	3.01E-04	3.389	0.0143	0.5139
GOBP_FAT	GO:0035295~tube development	19	3.55E-04	2.552	0.0165	0.6058
GOBP_FAT	GO:0048813~dendrite morphogenesis	21	3.99E-04	2.386	0.0181	0.6804
GOBP_FAT	GO:0016358~dendrite development	21	3.99E-04	2.386	0.0181	0.6804

GOBP_FAT	GO:0060446~branching involved in open tracheal system development	11	4.56E-04	3.750	0.0202	0.7783
GOBP_FAT	GO:0048754~branching morphogenesis of a tube	11	4.56E-04	3.750	0.0202	0.7783
GOBP_FAT	GO:0042067~establishment of ommatidial polarity	12	4.60E-04	3.468	0.0199	0.7847
GOBP_FAT	GO:0001736~establishment of planar polarity	14	4.82E-04	3.051	0.0204	0.8220
**GOBP_FAT**	**GO:0030166~proteoglycan biosynthetic process**	**7**	**4.86E-04**	**6.204**	**0.0201**	**0.8285**
GOBP_FAT	GO:0007164~establishment of tissue polarity	14	5.69E-04	3.002	0.0230	0.9696
**GOBP_FAT**	**GO:0006413~translational initiation**	**13**	**6.31E-04**	**3.142**	**0.0249**	**1.0738**
**GOBP_FAT**	**GO:0006029~proteoglycan metabolic process**	**7**	**7.29E-04**	**5.816**	**0.0282**	**1.2397**
GOBP_FAT	GO:0001763~morphogenesis of a branching structure	11	8.61E-04	3.482	0.0325	1.4628
GOBP_FAT	GO:0060562~epithelial tube morphogenesis	11	8.61E-04	3.482	0.0325	1.4628
**GOBP_FAT**	**GO:0030431~sleep**	**5**	**9.17E-04**	**9.496**	**0.0339**	**1.5576**
GOBP_FAT	GO:0007424~open tracheal system development	24	9.98E-04	2.085	0.0362	1.6948
GOBP_FAT	GO:0060541~respiratory system development	24	9.98E-04	2.085	0.0362	1.6948
**GOBP_FAT**	**GO:0006470~protein amino acid dephosphorylation**	**16**	**0.001011073**	**2.594**	**0.0360**	**1.7161**

GOBP_FAT	GO:0001737~establishment of imaginal disc-derived wing hair orientation	7	0.001055447	5.474	0.0369	1.7907
GOBP_FAT	GO:0007157~heterophilic cell adhesion	6	0.001191646	6.647	0.0408	2.0196
GOBP_FAT	GO:0048859~formation of anatomical boundary	11	0.00126658	3.324	0.0425	2.1453
GOBP_FAT	GO:0046530~photoreceptor cell differentiation	21	0.001290955	2.181	0.0426	2.1862
GOBP_FAT	GO:0060429~epithelium development	32	0.00139916	1.810	0.0453	2.3673
GOBP_FAT	GO:0007398~ectoderm development	14	0.00141723	2.737	0.0451	2.3976
GOBP_FAT	GO:0016203~muscle attachment	8	0.001453329	4.432	0.0455	2.4579
…						
**GOBP_FAT**	**GO:0007049~cell cycle**	**52**	**0.246263211**	**1.122**	**0.844**	**99.2074**
…						
**GOBP_FAT**	**GO:0048477~oogenesis**	**45**	**0.298595576**	**1.110**	**0.885**	**99.7686**
…						
**GOBP_FAT**	**GO:0006119~oxidative phosphorylation**	**11**	**0.536**	**1.108**	**0.972**	**99.9998**

1- DAVID analysis of genes with ≥ 1 polymorphism revealed most top-scoring Gene Ontology/Biological
Process (GOBP) annotations to relate to development and morphogenesis. Lower scoring annotations included 11 genes related to oxidative phosphorylation, 45 genes related to oogenesis and 52 to cell cycle, including ATR homolog, mei-41, with 26 SNPs, which regulates a meiotic checkpoint during Drosophila oogenesis.

Table S1C: Pathway Enrichment of AF Polymorphisms

Category	Term	Count	%	PValue	Genes	List Total	Pop Hits	Pop Total	Fold Enrichment
KEGG	dme04310:Wnt signaling pathway	15	1.419	0.002	FBGN0026597, FBGN0011655, FBGN0010015, FBGN0011826, FBGN0010333, FBGN0000119, FBGN0011817, FBGN0030505, FBGN0030758, FBGN0026174, FBGN0026181, FBGN0011577, FBGN0026175, FBGN0003371, FBGN0014020	170	74	2054	2.449
KEGG	dme00020:Citrate cycle (TCA cycle)	9	0.851	0.024	FBGN0035240, FBGN0038922, FBGN0030975, FBGN0035239, FBGN0029722, FBGN0027291, FBGN0052026, FBGN0028325, FBGN0036162, FBGN0001248	170	44	2054	2.471
KEGG	dme04650:Natural killer cell mediated cytotoxicity	6	0.568	0.030	FBGN0030758, FBGN0010015, FBGN0011826, FBGN0040068, FBGN0010333, FBGN0030505	170	22	2054	3.295
KEGG	dme04711:Circadian rhythm	4	0.378	0.043	FBGN0016694, FBGN0002413, FBGN0014396, FBGN0003371	170	10	2054	4.833
KEGG	dme04320:Dorso-ventral axis formation	6	0.568	0.057	FBGN0000256, FBGN0000810, FBGN0003731, FBGN0004647, FBGN0001404, FBGN0003118	170	26	2054	2.788
…									
KEGG	dme04350:TGF-beta signaling pathway	6	0.568	0.142	FBGN0026174, FBGN0026181, FBGN0011655, FBGN0003463, FBGN0026175, FBGN0014020	170	34	2054	2.132

KEGG	dme04070:Phosphatidylinositol signaling system	6	0.568	0.244	FBGN0051140, FBGN0085388, FBGN0085413, FBGN0085373, FBGN0030761, FBGN0040335	170	41	2054	1.768
KEGG	dme04150:mTOR signaling pathway	4	0.378	0.386	FBGN0010715, FBGN0015542, FBGN0035709, FBGN0035860	170	27	2054	1.790
KEGG	dme04013:MAPK signaling pathway	3	0.284	0.443	FBGN0003731, FBGN0003720, FBGN0003118	170	18	2054	2.014
KEGG	dme04330:Notch signaling pathway	2	0.189	0.850	FBGN0020388, FBGN0004647	170	22	2054	1.098
KEGG	dme04340:Hedgehog signaling pathway	2	0.189	0.874	FBGN0002413, FBGN0003371	170	24	2054	1.007
PANTHER	P00012:Cadherin signaling pathway	10	0.946	0.024	FBGN0000723, FBGN0011742, FBGN0001085, FBGN0046332, FBGN0003731, FBGN0003138, FBGN0001075, FBGN0003371, FBGN0000497, FBGN0039709	111	46	1160	2.272
PANTHER	P00057:Wnt signaling pathway	18	1.703	0.055	FBGN0259680, FBGN0043900, FBGN0001085, FBGN0046332, FBGN0011655, FBGN0000472, FBGN0010015, FBGN0011826, FBGN0001075, FBGN0011817, FBGN0000497, FBGN0052683, FBGN0020306, FBGN0025463, FBGN0002413, FBGN0030093, FBGN0003371, FBGN0039709	111	121	1160	1.555
PANTHER	P00007:Axon guidance mediated by semaphorins	5	0.473	0.057	FBGN0000723, FBGN0025743, FBGN0035574, FBGN0010333, FBGN0014020	111	16	1160	3.266

PANTHER	P00026:Heterotrimeric G-protein signaling pathway-Gi alpha and Gs alpha mediated pathway	9	0.851	0.084	FBGN0004573, FBGN0039747, FBGN0250910, FBGN0004834, FBGN0046332, FBGN0038063, FBGN0036789, FBGN0003371, FBGN0030087	111	49	1160	1.919
PANTHER	P00027:Heterotrimeric G-protein signaling pathway-Gq alpha and Go alpha mediated pathway	7	0.662	0.084	FBGN0039747, FBGN0030444, FBGN0259680, FBGN0053517, FBGN0035574, FBGN0036789, FBGN0014020	111	33	1160	2.217
…									
PANTHER	P00033:Insulin/IGF pathway-protein kinase B signaling cascade	4	0.378	0.621	FBGN0031086, FBGN0046332, FBGN0045759, FBGN0003371	111	33	1160	1.267
PANTHER	P00025:Hedgehog signaling pathway	3	0.284	0.744	FBGN0002413, FBGN0046332, FBGN0003371	111	27	1160	1.161
PANTHER	P00052:TGF-beta signaling pathway	4	0.378	0.916	FBGN0031086, FBGN0015789, FBGN0011655, FBGN0045759	111	56	1160	0.746
PANTHER	P00018:EGF receptor signaling pathway	4	0.378	0.932	FBGN0259680, FBGN0038603, FBGN0003731, FBGN0010333	111	59	1160	0.709
PANTHER	P00045:Notch signaling pathway	2	0.189	0.946	FBGN0020388, FBGN0004647	111	29	1160	0.721
