# Supplementary material for: Wnt Pathway Activation Increases Hypoxia Tolerance during Development
Source: PLoS One. 2014 Aug 5;9(8):e103292. doi: 10.1371/journal.pone.0103292 (PMC4122365; doi:10.1371/journal.pone.0103292)
Supplement: Table S2 — Wnt pathway polymorphisms. Table S2A: SNPs in Wnt-Pathway Associated Genes. Table S2B: Indels in Wnt-Pathway Associated Genes. Table S2C: Coding region Polymorphisms in Wnt pathway-Associated Genes. Table S2D: Polymorphism-Containing Wnt Pathway-Associated Genes. (PDF) [file pone.0103292.s006.pdf]

Table S2A: SNPs in Wnt-Pathway Associated Genes

SNP-Chrom		SNP-Location	FBgn	GeneName	Chrom	Start_Extended	End_Extended	Ref Base	Control Base	H-1 Base	H-2 Base
3R		25855268	FBgn0026597	Axn	3R	25846566	25863033	A	A	G	G
3R		25856260	FBgn0026597	Axn	3R	25846566	25863033	C	C	G	G
3R		25857098	FBgn0026597	Axn	3R	25846566	25863033	C	C	G	G
3R		25857108	FBgn0026597	Axn	3R	25846566	25863033	A	A	G	G
3R		25857149	FBgn0026597	Axn	3R	25846566	25863033	T	T	C	C
3R		25857899	FBgn0026597	Axn	3R	25846566	25863033	T	T	A	A
3R		25858547	FBgn0026597	Axn	3R	25846566	25863033	C	C	A	A
3L		1060268	FBgn0004870	bab1	3L	1034369	1103089	A	A	G	G
3L		1066588	FBgn0004870	bab1	3L	1034369	1103089	T	T	A	A
X	*	13051724	FBgn0025463	Bap60	X	13049478	13055611	C	C	T	T
X	*	13051921	FBgn0025463	Bap60	X	13049478	13055611	G	G	C	C
X	*	13051929	FBgn0025463	Bap60	X	13049478	13055611	G	G	A	A
3R		25678061	FBgn0039709	Cad99C	3R	25669326	25682425	C	C	T	T
3R	*	25678724	FBgn0039709	Cad99C	3R	25669326	25682425	T	T	C	C
3R	*	25680582	FBgn0039709	Cad99C	3R	25669326	25682425	A	A	T	T
3R	*	26868343	FBgn0010015	CanA1	3R	26863098	26874546	C	C	T	T
3R	*	26868364	FBgn0010015	CanA1	3R	26863098	26874546	T	T	C	C
3R	*	26868453	FBgn0010015	CanA1	3R	26863098	26874546	T	T	C	C
X		16468264	FBgn0030758	CanA-14F	X	16457506	16483160	G	G	T	T
X		16470744	FBgn0030758	CanA-14F	X	16457506	16483160	T	T	C	C
X		16471027	FBgn0030758	CanA-14F	X	16457506	16483160	T	T	G	G
X	*	16480873	FBgn0030758	CanA-14F	X	16457506	16483160	G	G	A	A
X	*	16480901	FBgn0030758	CanA-14F	X	16457506	16483160	G	G	A	A
X	*	16482157	FBgn0030758	CanA-14F	X	16457506	16483160	G	G	A	A
X	*	16482356	FBgn0030758	CanA-14F	X	16457506	16483160	G	G	A	A
X	*	16482494	FBgn0030758	CanA-14F	X	16457506	16483160	T	T	A	A
3R	*	17691971	FBgn0067317	Cby	3R	17691725	17696492	T	T	A	A
3R	*	17691989	FBgn0067317	Cby	3R	17691725	17696492	A	A	G	G

3R	*	17692079	FBgn0067317	Cby	3R	17691725	17696492	T	T	C	C
3R	*	17692121	FBgn0067317	Cby	3R	17691725	17696492	T	T	C	C
3R	*	17692411	FBgn0067317	Cby	3R	17691725	17696492	A	A	G	G
3R	*	17694243	FBgn0067317	Cby	3R	17691725	17696492	C	C	T	T
3L	*	6736271	FBgn0035713	CG10107	3L	6735526	6747386	G	G	T	T
3L	*	6736715	FBgn0035713	CG10107	3L	6735526	6747386	A	A	G	G
3L	*	6736925	FBgn0035713	CG10107	3L	6735526	6747386	C	C	T	T
3L	*	6736983	FBgn0035713	CG10107	3L	6735526	6747386	C	C	T	T
3L	*	6737096	FBgn0035713	CG10107	3L	6735526	6747386	A	A	T	T
3L	*	6737119	FBgn0035713	CG10107	3L	6735526	6747386	A	A	C	C
3L		6743650	FBgn0035713	CG10107	3L	6735526	6747386	C	C	G	G
3R		24898471	FBgn0039633	CG11873	3R	24888065	24934424	T	T	C	C
3R		24898541	FBgn0039633	CG11873	3R	24888065	24934424	A	A	G	G
3R		24898572	FBgn0039633	CG11873	3R	24888065	24934424	C	C	A	A
3R		24924998	FBgn0039633	CG11873	3R	24888065	24934424	G	G	C	C
3R		24926626	FBgn0039633	CG11873	3R	24888065	24934424	A	A	T	T
3R		24926645	FBgn0039633	CG11873	3R	24888065	24934424	G	G	A	A
3L	*	13373224	FBgn0036351	CG14107	3L	13371459	13376443	G	G	C	C
3L		12325640	FBgn0052105	CG32105	3L	12320336	12331006	G	G	C	C
X		16315878	FBgn0026575	hang	X	16314877	16333531	T	T	C	C
X		16315939	FBgn0026575	hang	X	16314877	16333531	G	G	T	T
X		16318068	FBgn0026575	hang	X	16314877	16333531	A	A	G	G
X		16318530	FBgn0026575	hang	X	16314877	16333531	G	G	T	T
X		16318819	FBgn0026575	hang	X	16314877	16333531	G	G	C	C
X		16318865	FBgn0026575	hang	X	16314877	16333531	A	A	G	G
X		16318943	FBgn0026575	hang	X	16314877	16333531	C	C	G	G
X		16318984	FBgn0026575	hang	X	16314877	16333531	G	G	C	C
X		16318993	FBgn0026575	hang	X	16314877	16333531	T	T	C	C
X		16319584	FBgn0026575	hang	X	16314877	16333531	C	C	T	T
X		16320246	FBgn0026575	hang	X	16314877	16333531	T	T	A	A
X		16321056	FBgn0026575	hang	X	16314877	16333531	C	C	T	T

X		16321537	FBgn0026575	hang	X	16314877	16333531	T	T	C	C
X		16322274	FBgn0026575	hang	X	16314877	16333531	C	C	G	G
X		16322483	FBgn0026575	hang	X	16314877	16333531	A	A	G	G
X		16323014	FBgn0026575	hang	X	16314877	16333531	T	T	G	G
X		16323338	FBgn0026575	hang	X	16314877	16333531	C	C	T	T
X		16323466	FBgn0026575	hang	X	16314877	16333531	A	A	C	C
X		16323533	FBgn0026575	hang	X	16314877	16333531	T	T	A	A
X		16323941	FBgn0026575	hang	X	16314877	16333531	A	A	T	T
X		16324767	FBgn0026575	hang	X	16314877	16333531	C	C	T	T
X		16324823	FBgn0026575	hang	X	16314877	16333531	G	G	C	C
X		16324886	FBgn0026575	hang	X	16314877	16333531	C	C	A	A
X		16324923	FBgn0026575	hang	X	16314877	16333531	T	T	C	C
X		16325000	FBgn0026575	hang	X	16314877	16333531	T	T	C	C
X		16325027	FBgn0026575	hang	X	16314877	16333531	G	G	A	A
X		16325138	FBgn0026575	hang	X	16314877	16333531	A	A	G	G
X		16325270	FBgn0026575	hang	X	16314877	16333531	G	G	A	A
X		16325480	FBgn0026575	hang	X	16314877	16333531	A	A	G	G
X		16325894	FBgn0026575	hang	X	16314877	16333531	T	T	C	C
X		16325939	FBgn0026575	hang	X	16314877	16333531	C	C	T	T
X		16325942	FBgn0026575	hang	X	16314877	16333531	A	A	G	G
X		16325975	FBgn0026575	hang	X	16314877	16333531	T	T	C	C
X		16326152	FBgn0026575	hang	X	16314877	16333531	C	C	T	T
X		16326182	FBgn0026575	hang	X	16314877	16333531	G	G	A	A
X		16326212	FBgn0026575	hang	X	16314877	16333531	A	A	G	G
X		16326458	FBgn0026575	hang	X	16314877	16333531	C	C	T	T
X		16327253	FBgn0026575	hang	X	16314877	16333531	T	T	C	C
X		16327985	FBgn0026575	hang	X	16314877	16333531	T	T	C	C
X		16327994	FBgn0026575	hang	X	16314877	16333531	A	A	T	T
X		16328249	FBgn0026575	hang	X	16314877	16333531	G	G	T	T
X		16328265	FBgn0026575	hang	X	16314877	16333531	A	A	C	C
X		16329558	FBgn0026575	hang	X	16314877	16333531	G	G	A	A
X		16329702	FBgn0026575	hang	X	16314877	16333531	C	C	A	A

X		16329714	FBgn0026575	hang	X	16314877	16333531	A	A	G	G
X		16329972	FBgn0026575	hang	X	16314877	16333531	C	C	T	T
X		16330252	FBgn0026575	hang	X	16314877	16333531	A	A	C	C
X	*	16333134	FBgn0026575	hang	X	16314877	16333531	G	G	A	A
X	*	16333145	FBgn0026575	hang	X	16314877	16333531	A	A	G	G
3L		12284712	FBgn0036274	CG4328	3L	12282056	12291121	A	A	C	C
X		18537604	FBgn0027335	Rip11	X	18527667	18543596	T	T	C	C
3R	*	7514334	FBgn0037935	CG6834	3R	7508391	7515852	G	G	T	T
3L		11120635	FBgn0260795	CG42575	3L	11114461	11129763	T	T	C	C
3L		11120643	FBgn0260795	CG42575	3L	11114461	11129763	T	T	G	G
3L	*	11126284	FBgn0260795	CG42575	3L	11114461	11129763	C	C	T	T
3R	*	25621714	FBgn0039696	CG7837	3R	25619419	25627055	T	T	C	C
3R	*	25621888	FBgn0039696	CG7837	3R	25619419	25627055	A	A	G	G
3R	*	25623948	FBgn0039696	CG7837	3R	25619419	25627055	A	A	G	G
3R	*	25623950	FBgn0039696	CG7837	3R	25619419	25627055	A	A	C	C
3R	*	25624104	FBgn0039696	CG7837	3R	25619419	25627055	A	A	G	G
3R	*	25624556	FBgn0039696	CG7837	3R	25619419	25627055	T	T	A	A
3R	*	25625952	FBgn0039696	CG7837	3R	25619419	25627055	T	T	C	C
3R	*	13825334	FBgn0000363	cpo	3R	13755595	13843501	G	G	A	A
X	*	9047536	FBgn0030093	dalao	X	9041358	9048011	T	T	C	C
X	*	9047809	FBgn0030093	dalao	X	9041358	9048011	G	G	A	A
3R		26882410	FBgn0002413	dco	3R	26878905	26888931	G	G	A	A
3R		26882515	FBgn0002413	dco	3R	26878905	26888931	T	T	A	A
3R		26882761	FBgn0002413	dco	3R	26878905	26888931	T	T	C	C
3R		26882794	FBgn0002413	dco	3R	26878905	26888931	G	G	A	A
3R		26886394	FBgn0002413	dco	3R	26878905	26888931	T	T	A	A
X		3278089	FBgn0000472	dm	X	3265216	3282049	G	G	A	A
2R	*	17219902	FBgn0020306	dom	2R	17208949	17231352	T	T	C	C
2R	*	17220640	FBgn0020306	dom	2R	17208949	17231352	C	C	T	T
2L		659638	FBgn0000497	ds	2L	638021	716968	T	T	C	C
2L		4214127	FBgn0001075	ft	2L	4196402	4219852	T	T	G	G

2L		4214130	FBgn0001075	ft	2L	4196402	4219852	A	A	T	T	
3L		14299897	FBgn0001085	fz	3L	14265447	14363748	T	T	G	G	
3R	*	27196173	FBgn0046332	gskt	3R	27196149	27201920	C	C	G	G	
3R		26156142	FBgn0010113	hdc	3R	26101656	26189891	T	T	G	G	
3R		26157633	FBgn0010113	hdc	3R	26101656	26189891	G	G	C	C	
3R		26174967	FBgn0010113	hdc	3R	26101656	26189891	T	T	A	A	
3R		26177602	FBgn0010113	hdc	3R	26101656	26189891	A	A	G	G	
3R		26184449	FBgn0010113	hdc	3R	26101656	26189891	C	C	T	T	
3R		26184459	FBgn0010113	hdc	3R	26101656	26189891	C	C	T	T	
X		13543214	FBgn0030505	NFAT	X	13516790	13559690	C	C	T	T	
3L		7976664	FBgn0011817	nmo	3L	7970149	8045450	C	C	T	T	
3L	*	8023055	FBgn0011817	nmo	3L	7970149	8045450	C	C	T	T	
3L	*	8023278	FBgn0011817	nmo	3L	7970149	8045450	A	A	G	G	
3L		8030970	FBgn0011817	nmo	3L	7970149	8045450	G	G	A	A	
3L		8032191	FBgn0011817	nmo	3L	7970149	8045450	G	G	A	A	
3L		8035281	FBgn0011817	nmo	3L	7970149	8045450	T	T	C	C	
3L		8044386	FBgn0011817	nmo	3L	7970149	8045450	T	T	A	A	
3L		8044395	FBgn0011817	nmo	3L	7970149	8045450	A	A	T	T	
X		12324763	FBgn0259680	Pkcdelta	X	12324259	12349683	C	C	A	A	
X		12344985	FBgn0259680	Pkcdelta	X	12324259	12349683	G	G	C	C	
3R		19126196	FBgn0003118	pnt	3R	19114826	19173884	T	T	C	C	
3R		19169700	FBgn0003118	pnt	3R	19114826	19173884	C	C	T	T	
3R		19169703	FBgn0003118	pnt	3R	19114826	19173884	A	A	C	C	
3R		19171816	FBgn0003118	pnt	3R	19114826	19173884	A	A	T	T	
3R	*	19171970	FBgn0003118	pnt	3R	19114826	19173884	T	T	C	C	
3R	*	19173445	FBgn0003118	pnt	3R	19114826	19173884	C	C	G	G	
X		16451640	FBgn0011826	Pp2B-14D	X	16448797	16456052	C	C	T	T	
X		16451663	FBgn0011826	Pp2B-14D	X	16448797	16456052	C	C	G	G	
X		16453000	FBgn0011826	Pp2B-14D	X	16448797	16456052	A	A	G	G	
X		16453114	FBgn0011826	Pp2B-14D	X	16448797	16456052	A	A	G	G	
X		16453381	FBgn0011826	Pp2B-14D	X	16448797	16456052	G	G	A	A	
X		16453645	FBgn0011826	Pp2B-14D	X	16448797	16456052	G	G	A	A	

X		16453711	FBgn0011826	Pp2B-14D	X	16448797	16456052	C	C	T	T
3R		27403752	FBgn0043900	pygo	3R	27399557	27408412	A	A	T	T
3L	*	1300237	FBgn0010333	Rac1	3L	1298879	1304683	G	G	A	A
3L	*	1303255	FBgn0010333	Rac1	3L	1298879	1304683	A	A	G	G
X		16522505	FBgn0026181	rok	X	16517142	16533452	C	C	T	T
X	*	16523825	FBgn0026181	rok	X	16517142	16533452	A	A	G	G
X	*	16530148	FBgn0026181	rok	X	16517142	16533452	G	G	T	T
X	*	16530173	FBgn0026181	rok	X	16517142	16533452	G	G	A	A
X	*	16530435	FBgn0026181	rok	X	16517142	16533452	T	T	C	C
X	*	16530805	FBgn0026181	rok	X	16517142	16533452	G	G	C	C
X	*	16530963	FBgn0026181	rok	X	16517142	16533452	T	T	G	G
X	*	16531499	FBgn0026181	rok	X	16517142	16533452	A	A	C	C
X	*	16531593	FBgn0026181	rok	X	16517142	16533452	G	G	A	A
X	*	16531595	FBgn0026181	rok	X	16517142	16533452	A	A	C	C
X	*	16532133	FBgn0026181	rok	X	16517142	16533452	G	G	C	C
X	*	16532262	FBgn0026181	rok	X	16517142	16533452	G	G	A	A
X	*	16532401	FBgn0026181	rok	X	16517142	16533452	C	C	T	T
X	*	16532402	FBgn0026181	rok	X	16517142	16533452	C	C	A	A
3L		6532545	FBgn0020251	sfl	3L	6486830	6544346	A	A	G	G
3L		6534030	FBgn0020251	sfl	3L	6486830	6544346	A	A	G	G
3L		6534065	FBgn0020251	sfl	3L	6486830	6544346	T	T	G	G
3L		6540733	FBgn0020251	sfl	3L	6486830	6544346	C	C	G	G
X		2530444	FBgn0003371	sgg	X	2525983	2573890	T	T	G	G
X		2532290	FBgn0003371	sgg	X	2525983	2573890	A	A	C	C
X		2553538	FBgn0003371	sgg	X	2525983	2573890	C	C	T	T
X		2554975	FBgn0003371	sgg	X	2525983	2573890	T	T	C	C
X		2560726	FBgn0003371	sgg	X	2525983	2573890	G	G	A	A
3R	*	25883225	FBgn0015542	sima	3R	25882036	25949113	T	T	C	C
3R	*	25888163	FBgn0015542	sima	3R	25882036	25949113	A	A	G	G
3R	*	25890435	FBgn0015542	sima	3R	25882036	25949113	C	C	G	G
3R		25894002	FBgn0015542	sima	3R	25882036	25949113	A	A	G	G

3R		25894059	FBgn0015542	sima	3R	25882036	25949113	C	C	T	T
3R		25897834	FBgn0015542	sima	3R	25882036	25949113	C	T	A	A
3R		25897835	FBgn0015542	sima	3R	25882036	25949113	G	G	T	T
3R		25920200	FBgn0015542	sima	3R	25882036	25949113	G	G	A	A
3R		25925062	FBgn0015542	sima	3R	25882036	25949113	T	T	C	C
3R		25925100	FBgn0015542	sima	3R	25882036	25949113	T	T	C	C
3R		25928248	FBgn0015542	sima	3R	25882036	25949113	T	T	G	G
3R		25934509	FBgn0015542	sima	3R	25882036	25949113	C	C	T	T
3R		25940704	FBgn0015542	sima	3R	25882036	25949113	A	A	T	T
3R	*	25948527	FBgn0015542	sima	3R	25882036	25949113	C	C	G	G
X		19711825	FBgn0026175	skpC	X	19708171	19712647	G	G	A	A
X		19705790	FBgn0026174	skpD	X	19704762	19709421	T	T	C	C
X		12584535	FBgn0024308	Smr	X	12578031	12638476	T	T	C	C
X		12584544	FBgn0024308	Smr	X	12578031	12638476	T	T	C	C
X		12584820	FBgn0024308	Smr	X	12578031	12638476	T	T	C	C
X		12584838	FBgn0024308	Smr	X	12578031	12638476	A	A	G	G
X		12584850	FBgn0024308	Smr	X	12578031	12638476	C	C	A	A
X		12586881	FBgn0024308	Smr	X	12578031	12638476	T	T	C	C
X		12594631	FBgn0024308	Smr	X	12578031	12638476	C	C	G	G
X	*	12600108	FBgn0024308	Smr	X	12578031	12638476	T	T	C	C
X	*	12600167	FBgn0024308	Smr	X	12578031	12638476	C	C	T	T
X	*	12601629	FBgn0024308	Smr	X	12578031	12638476	T	T	C	C
X	*	12601631	FBgn0024308	Smr	X	12578031	12638476	C	C	A	A
X	*	12603364	FBgn0024308	Smr	X	12578031	12638476	T	T	G	G
X	*	12603450	FBgn0024308	Smr	X	12578031	12638476	A	A	G	G
X		12612904	FBgn0024308	Smr	X	12578031	12638476	A	A	C	C
X		12621985	FBgn0024308	Smr	X	12578031	12638476	G	G	C	C
3L		10853267	FBgn0026160	tna	3L	10826628	10869762	A	A	C	C
3L		5838466	FBgn0003984	vn	3L	5804438	5840764	G	G	A	A
3R		26618196	FBgn0011739	wts	3R	26613379	26634341	C	C	T	T

Column header abbreviations: *SNP Chrom* - Chromosome on which the SNP occurs; *SNP Location* - Locus at which the SNP occurs; *FBgn* - FlyBase gene identifier (number); *Chrom* - Same as SNP Chrom; *Start_Extended* - Locus at which the extended gene begins (FlyBase release 5.16); *End_Extended* - Locus at which the extended gene ends (FlyBase release 5.16); *Ref Base* - Nucleotide at this locus in the D. melanogaster Reference Genome; *Control Base* - Nucleotide at this locus in the Control (C-1) genome; *H-1Base* - Nucleotide at this locus in the hypoxia-adapted genome (H-1); *H-2Base* - Nucleotide at this locus in the hypoxia-adapted genome (H-2).

* - Locus also maps to other, non-Wnt associated gene(s); this occurs due to close proximity/overlap of genes in the D. melanogaster genome and to our evaluation of extended genes encompassing multiple gene regions.

Table S2B: Indels in Wnt-Pathway Associated Genes

Indel-Chrom		Indel-Location	FBgn	GeneName	Chrom	Start_Extended	End_Extended	H-1 Indel Size	H-2 Indel Size
2R		9361436	FBgn0000119	arr	2R	9339138	9372723	-4	-4
3L		1067902	FBgn0004870	bab1	3L	1034369	1103089	-12	-12
3R	*	16419218	FBgn0023097	bon	3R	16416881	16440262	7	7
3R	*	16419269	FBgn0023097	bon	3R	16416881	16440262	-3	-3
X	*	16480046	FBgn0030758	CanA-14F	X	16457506	16483160	1	1
3L	*	6736946	FBgn0035713	CG10107	3L	6735526	6747386	-1	-1
3L		20843272	FBgn0052432	CG32432	3L	20835425	20880288	-1	-1
X		16324021	FBgn0026575	hang	X	16314877	16333531	-3	-3
X	*	10310448	FBgn0052683	CG32683	X	10302420	10350928	1	1
3L		11120212	FBgn0260795	CG42575	3L	11114461	11129763	1	1
X		19092498	FBgn0030997	CG7990	X	19088091	19097810	1	1
3L	*	8833831	FBgn0011577	dally	3L	8818606	8885269	1	1
3R		26881704	FBgn0002413	dco	3R	26878905	26888931	-1	-1
3R		14292859	FBgn0004652	fru	3R	14240315	14373308	1	1
3R		26152119	FBgn0010113	hdc	3R	26101656	26189891	1	1
3R		26155075	FBgn0010113	hdc	3R	26101656	26189891	-1	-1
2L		2918409	FBgn0041111	lilli	2L	2883952	2955077	-4	-4
3R	*	27437643	FBgn0011655	Med	3R	27434770	27443478	2	2
3L		8030913	FBgn0011817	nmo	3L	7970149	8045450	1	1
X		16454010	FBgn0011826	Pp2B-14D	X	16448797	16456052	1	1
2R	*	11992045	FBgn0014020	Rho1	2R	11988191	11996734	-2	-1
X	*	16530163	FBgn0026181	rok	X	16517142	16533452	-3	-3
X	*	16531620	FBgn0026181	rok	X	16517142	16533452	2	2
X	*	16531813	FBgn0026181	rok	X	16517142	16533452	1	1
X	*	16532424	FBgn0026181	rok	X	16517142	16533452	-1	-1
3L		6531103	FBgn0020251	sfl	3L	6486830	6544346	-1	-1
X		2538552	FBgn0003371	sgg	X	2525983	2573890	1	1
3L	*	6957020	FBgn0010851	sgl	3L	6950647	6959902	-3	-3

3R	*	25890695	FBgn0015542	sima	3R	25882036	25949113	-1	-1
3R		25921624	FBgn0015542	sima	3R	25882036	25949113	-1	-1
3R		25922644	FBgn0015542	sima	3R	25882036	25949113	-2	-2
3R		25925309	FBgn0015542	sima	3R	25882036	25949113	-1	-1
3R		25928578	FBgn0015542	sima	3R	25882036	25949113	-3	-3
X		19711833	FBgn0026175	skpC	X	19708171	19712647	2	2
3L		10858407	FBgn0026160	tna	3L	10826628	10869762	-1	-1
2R		10428290	FBgn0020245	ttv	2R	10412076	10474826	-1	-1
3R		26628332	FBgn0011739	wts	3R	26613379	26634341	4	4

Column header abbreviations: *Indel Chrom* - Chromosome on which the Indel occurs; *Indel Location* - Locus at which the Indel occurs; *FBgn* - FlyBase gene identifier (number); *Chrom* - Same as SNP Chrom; *Start_Extended* - Locus at which the extended gene begins (FlyBase release 5.16); *End_Extended* - Locus at which the extended gene ends (FlyBase release 5.16); *Ref Base* - Nucleotide at this locus in the D. melanogaster Reference Genome; *Control Base* - Nucleotide at this locus in the Control (C-1) genome; *H-1Base* - Nucleotide at this locus in the hypoxia-adapted genome (H-1); *H-2Base* - Nucleotide at this locus in the hypoxia-adapted genome (H-2).

* - Locus also maps to other, non-Wnt associated gene(s); this occurs due to close proximity/overlap of genes in the D. melanogaster genome and to our evaluation of extended genes encompassing multiple gene regions.

Table S2C: Coding region Polymorphisms in Wnt pathway-Associated Genes

FBgn	SNP-Chrom	SNP-Location	GeneName	Strand	Frame	Ref-Codon	Ref-AmAc	SNP-Codon	SNP-AmAc	Type
FBgn0026597	3R	25857149	Axn	+	1	TTG	L	CTG	L	Synonymous
FBgn0039709	3R	25678061	Cad99C	+	0	GAC	D	GAT	D	Synonymous
FBgn0039709	3R	25678724	Cad99C	+	0	CGT	R	CGC	R	Synonymous
FBgn0010015	3R	26868343	CanA1	+	0	AGC	S	AGT	S	Synonymous
FBgn0010015	3R	26868364	CanA1	+	0	TTT	F	TTC	F	Synonymous
FBgn0067317	3R	17694243	Cby	+	0	CTC	L	CTT	L	Synonymous
FBgn0039633	3R	24924998	CG11873	+	0	AGC	S	ACC	T	Non-Synonymous
FBgn0039633	3R	24926626	CG11873	+	0	ACT	T	TCT	S	Non-Synonymous
FBgn0039633	3R	24926645	CG11873	+	0	AGC	S	AAC	N	Non-Synonymous
FBgn0026575	X	16323338	hang	+	0	CTC	L	CTT	L	Synonymous
FBgn0026575	X	16323466	hang	+	0	CAA	Q	CCA	P	Non-Synonymous
FBgn0026575	X	16323533	hang	+	0	GCT	A	GCA	A	Synonymous
FBgn0026575	X	16323941	hang	+	0	ACA	T	ACT	T	Synonymous
FBgn0026575	X	16324767	hang	+	0	CGC	R	CGT	R	Synonymous
FBgn0026575	X	16325000	hang	+	0	GCT	A	GCC	A	Synonymous
FBgn0026575	X	16325027	hang	+	0	CCG	P	CCA	P	Synonymous
FBgn0026575	X	16325138	hang	+	0	CTA	L	CTG	L	Synonymous
FBgn0026575	X	16325270	hang	+	0	CGG	R	CGA	R	Synonymous
FBgn0026575	X	16325480	hang	+	0	AAA	K	AAG	K	Synonymous
FBgn0026575	X	16325894	hang	+	0	ACT	T	ACC	T	Synonymous
FBgn0026575	X	16325939	hang	+	0	GTC	V	GTT	V	Synonymous
FBgn0026575	X	16325942	hang	+	0	CAA	Q	CAG	Q	Synonymous
FBgn0026575	X	16325975	hang	+	0	TTT	F	TTC	F	Synonymous
FBgn0026575	X	16326152	hang	+	0	AAC	N	AAT	N	Synonymous
FBgn0026575	X	16326182	hang	+	0	AGG	R	AGA	R	Synonymous
FBgn0026575	X	16326212	hang	+	0	AAA	K	AAG	K	Synonymous
FBgn0026575	X	16326458	hang	+	0	AAC	N	AAT	N	Synonymous
FBgn0026575	X	16327253	hang	+	2	AGT	S	AGC	S	Synonymous

FBgn0026575	X	16327985	hang	+	2	TCT	S	TCC	S	Synonymous
FBgn0026575	X	16327994	hang	+	2	GCA	A	GCT	A	Synonymous
FBgn0026575	X	16329558	hang	+	0	GAG	E	GAA	E	Synonymous
FBgn0026575	X	16329702	hang	+	0	GTC	V	GTA	V	Synonymous
FBgn0026575	X	16329714	hang	+	0	GTA	V	GTG	V	Synonymous
FBgn0026575	X	16329972	hang	+	0	GAC	D	GAT	D	Synonymous
FBgn0026575	X	16330252	hang	+	0	ACG	T	CCG	P	Non-Synonymous
FBgn0260795	3L	11126284	CG42575	+	0	AGC	S	AGT	S	Synonymous
FBgn0039696	3R	25621714	CG7837	-	0	GAA	E	GAG	E	Synonymous
FBgn0039696	3R	25621888	CG7837	-	0	CAT	H	CAC	H	Synonymous
FBgn0039696	3R	25624104	CG7837	-	0	TTT	F	TTC	F	Synonymous
FBgn0002413	3R	26882410	dco	-	0	GAC	D	GAT	D	Synonymous
FBgn0002413	3R	26882515	dco	-	0	GCA	A	GCT	A	Synonymous
FBgn0002413	3R	26882761	dco	-	0	AAA	K	AAG	K	Synonymous
FBgn0002413	3R	26882794	dco	-	0	TTC	F	TTT	F	Synonymous
FBgn0020306	2R	17219902	dom	+	0	GGT	G	GGC	G	Synonymous
FBgn0020306	2R	17220640	dom	+	0	CAC	H	CAT	H	Synonymous
FBgn0001075	2L	4214127	ft	-	0	TCA	S	TCC	S	Synonymous
FBgn0001075	2L	4214130	ft	-	0	GGT	G	GGA	G	Synonymous
FBgn0011826	X	16453000	Pp2B-14D	-	0	ATT	I	ATC	I	Synonymous
FBgn0011826	X	16453114	Pp2B-14D	-	0	CAT	H	CAC	H	Synonymous
FBgn0011826	X	16453381	Pp2B-14D	-	0	GTC	V	GTT	V	Synonymous
FBgn0043900	3R	27403752	pygo	-	0	ATC	I	AAC	N	Non-Synonymous
FBgn0026181	X	16522505	rok	-	0	GAG	E	GAA	E	Synonymous
FBgn0026181	X	16523825	rok	-	0	GGT	G	GGC	G	Synonymous
FBgn0003371	X	2553538	sgg	+	0	AGC	S	AGT	S	Synonymous
FBgn0003371	X	2554975	sgg	+	0	GCT	A	GCC	A	Synonymous
FBgn0015542	3R	25894059	sima	+	1	TCG	S	TTG	L	Non-Synonymous
FBgn0024308	X	12584535	Smr	-	2	CAA	Q	CAG	Q	Synonymous
FBgn0024308	X	12584544	Smr	-	2	CAA	Q	CAG	Q	Synonymous
FBgn0024308	X	12584820	Smr	-	2	CCA	P	CCG	P	Synonymous
FBgn0024308	X	12584838	Smr	-	2	GGT	G	GGC	G	Synonymous

FBgn0024308	X	12584850	Smr	-	2	TCG	S	TCT	S	Synonymous
FBgn0024308	X	12586881	Smr	-	2	CAA	Q	CAG	Q	Synonymous
FBgn0011739	3R	26618196	wts	-	0	AAG	K	AAA	K	Synonymous

Column header abbreviations: *FBgn* - FlyBase gene identifier (number); *SNP Chrom* - Chromosome on which the SNP occurs; *SNP Locatio*n - Locus at which the SNP occurs; *Ref-Codon* - Codon in the D. melanogaster Reference Genome containing the SNP location identified in the hypoxia-adapted genome; *Ref-AmAc* - Corresponding amino acid translation of the D. melanogaster Reference codon; *SNP-Codon* - Codon in the hypoxia-adapted genome containing the identified SNP; *SNP-AmAc* - Corresponding amino acid translation of the hypoxia-adapted codon

Table S2D: Polymorphism-Containing Wnt Pathway-Associated Genes.

Symbol[1]	Enrichment Source[2]	Wnt Pathway	Function[2]	SNP	Indel	Polymorph/kB[3]	Number CDS SNP	Number NS SNP
arr	KEGG, DasGupta, GO	Canonical	Co-Receptor; RNAi: Activator		1	0.030		
Axn	KEGG, DasGupta, GO	Canonical	β-catenin binding; RNAi: Inhibitor	7		0.425	1	
bab1	DasGupta	Canonical	RNAi:Inhibitor	2	1	0.044		
Bap60	Panther	Canonical	Brahma associated protein; Activator	3		0.489		
bon	DasGupta	Canonical	RNAi:Inhibitor		2	0.086		
Cad99C	Panther		Cadherin	3		0.176	2	
CanA1	KEGG, Panther	Calcium	Dephosphorylates NFAT; Activator	3		0.262	2	
CanA-14F	KEGG	Calcium	Dephosphorylates NFAT; Activator	8	1	0.331		
Cby	GO	Canonical	Inhibitor	6		1.258	1	
CG10107	DasGupta	Canonical	RNAi:Inhibitor	7	1	0.674		
CG11873	DasGupta	Canonical	RNAi:Inhibitor	6		0.129	3	3
CG14107	DasGupta	Canonical	RNAi:Activator	1		0.201		
CG32105	DasGupta	Canonical	RNAi:Inhibitor	1		0.094		
CG32432	DasGupta	Canonical	RNAi:Inhibitor		1	0.022		
CG32575/hang	DasGupta	Canonical	RNAi:Inhibitor	49	1	2.680	26	2
CG32683	Panther	Canonical	Arrestin-related; β-arrestin is an Activator		1	0.021		
CG4328	DasGupta	Canonical	RNAi:Inhibitor	1		0.110		
CG6606/Rip11	DasGupta	Canonical	RNAi:Activator	1		0.063		
CG6834	DasGupta	Canonical	RNAi:Activator	1		0.134		
CG7628/CG42575	DasGupta	Canonical	RNAi:Inhibitor	3	1	0.261	1	
CG7837	DasGupta	Canonical	RNAi:Activator	7		0.917	3	

CG7990	DasGupta	Canonical	RNAi:Inhibitor		1	0.103		
cpo	DasGupta	Canonical	RNAi:Inhibitor	1		0.010		
dalao	Panther	Canonical	Transcription regulation; Activator	2		0.301		
dally	KEGG, GO	Canonical	Co-Receptor		1	0.015		
dco	Panther	Canonical	Casein kinase Iε; Activator	5	1	0.598	4	
dm	Panther	Canonical	(Myc) Transcription factor; Target Gene	1		0.059		
dom	Panther, DasGupta	Canonical	Helicase; RNAi:Inhibitor	2		0.089	2	
ds	Panther	PCP	Cadherin-related	1		0.013		
fru	DasGupta	Canonical	RNAi:Inhibitor		1	0.008		
ft	Panther, GO	PCP	Cadherin-related	2		0.085	2	
fz	Panther, DasGupta, GO	PCP > Canonical	Receptor; RNAi: Activator	1		0.010		
gskt	Panther	Canonical	Putative GSK3Bhomolog; Inhibitor	1		0.173		
hdc	DasGupta	Canonical	RNAi:Inhibitor	6	2	0.091		
lilli	DasGupta	Canonical	RNAi:Activator		1	0.014		
Med	KEGG, Panther	Canonical	Binds TCF; Activator		1	0.123		
NFAT	KEGG	Calcium	Transcription factor; Activator	1		0.023		
nmo	KEGG, Panther, GO	Canonical	Kinase; Inhibitor	8	1	0.120		
Pkcdelta	Panther	Calcium	Kinase	2		0.079		
pnt	DasGupta	Canonical	RNAi:Inhibitor	6		0.102		
Pp2B-14D	KEGG, Panther	Calcium	Dephosphorylates NFAT; Activator	7	1	0.935	3	
pygo	Panther, DasGupta,	Canonical	β-catenin nuclear targeting;	1		0.113	1	1

	GO		RNAi:Activator					
Rac1	KEGG	PCP	Activator	2		0.345		
Rho1	KEGG, GO	PCP	Activator		1	0.117		
rok	KEGG, GO	PCP	Activator	14	4	1.104	2	
sfl	GO	Canonical	Sulfotransferase; Activator	4	1	0.087		
sgg	KEGG, Panther, GO	Canonical	GSK3B homolog; β-catenin binding, Inhibitor	5	1	0.125	2	
sgl	GO	Canonical	UDP Glucose Dehydrogenase; Activator		1	0.108		
sima	DasGupta	Canonical	RNAi:Inhibitor	14	5	0.283	1	1
skpC	KEGG	Canonical	β-catenin proteolysis; Inhibitor	1	1	0.447		
skpD	KEGG	Canonical	β-catenin proteolysis; Inhibitor	1		0.215		
Smr	DasGupta	Canonical	RNAi:Inhibitor	15		0.248	6	
tna	DasGupta	Canonical	RNAi:Inhibitor	1	1	0.046		
ttv	GO		Heparan sulfate proteoglycan biosynthesis; Activator		1	0.016		
vn	DasGupta	Canonical	RNAi:Inhibitor	1		0.028		
wts	DasGupta	Canonical	RNAi:Inhibitor	1	1	0.095	1	

Column header abbreviations: *Number CDS SNP* - Number of coding sequence SNPs; *Number NS SNP* - Number of non-synonymous coding sequence SNPs

1-Reflecting gene overlap in the Drosophila genome, some mutations also map to other genes.
2-Pathway and function information was compiled using KEGG, Panther, DasGupta, GO and FlyBase sources (references 14,15; 16; 18; 17; and 10, respectively, in the manuscript).
3-Polymorphisms/kB were calculated for extended genes: gene plus 2kB up- and downstream.
