# Supplementary material for: Wnt Pathway Activation Increases Hypoxia Tolerance during Development
Source: PLoS One. 2014 Aug 5;9(8):e103292. doi: 10.1371/journal.pone.0103292 (PMC4122365; doi:10.1371/journal.pone.0103292)
Supplement: Table S3 — Differential Gene Expression. Table S3A: KEGG and GO BP annotations for DE genes shared by H and HR. Table S3B: Pathway Analysis of Differential Expression in Post-Eclosion Hypoxia-Tolerant Flies Maintained in 4% O2. (PDF) [file pone.0103292.s007.pdf]

Table S3A: KEGG and GO BP annotations for DE genes shared by H and HR

	Adult		Post-Eclosion		Larva	
	Annotation	Number	Annotation	Number	Annotation	Number
**UPREGULATED**						
KEGG	*Limonene/pinene degradation*	5	*Limonene/pinene degradation*	3	*Metabolism xenobiotics (P450)*	5
	Lysosome	3			*Drug metabolism*	5
	Glutathione metabolism	2			Tyrosine metabolism	3
	Metabolism xenobiotics (P450)	2			Retinol metabolism	3
	Drug metabolism	2			Fatty acid metabolism	2
GO-BP	*Defense response*	10	Sperm individualization	2	Oxidation reduction	11
	…				Proteolysis	10
	Antimicrobial humoral response	5			*Defense response*	4
**DOWNREGULATED**						
KEGG	*Limonene/pinene degradation*	4	*Limonene/pinene degradation*	2	*Limonene/pinene degradation*	4
	Metabolism xenobiotics (P450)	3			Glycerophospholipid metab	2
	Drug metabolism	3			*Glutathione metabolism*	2
	Lysosome	3			*Metabolism xenobiotics (P450)*	2
	…				Drug metabolism	2
	Starch & sucrose metabolism	2				
	Glutathione metabolism	2				
GO-BP	Peptidoglycan metabolic process	4	Chorion-containing eggshell formation	6	*Oxidation reduction*	11
	…		…		*Defense response*	6
	Oxidation reduction	18	Sexual reproduction / Oogenesis	7	Biogenic amine metabolic process	3
	Defense response	8	…		Locomotory behavior	5
			Oxidation reduction	6		
			Proteolysis	6		
			Defense response to fungus	2		

Table S3B: Pathway Analysis of Differential Expression in Post-Eclosion Hypoxia-Tolerant Flies Maintained in 4% O2

Category	Term[1]	Count[2]	%	PValue	Genes	List Total	Pop Hits	Pop Total	Fold Enrichment
KEGG	dme04340:Hedgehog signaling pathway	9	0.230888	0.549214	FBgn0000352, FBgn0250823, FBgn0000274, FBgn0003444, FBgn0037364, FBgn0003371, FBgn0001079, FBgn0085381, FBgn0004859	672	24	2054	1.146205357
KEGG	dme04310:Wnt signaling pathway	23	0.590046	0.747699	FBgn0004624, FBgn0010315, FBgn0003091, FBgn0000259, FBgn0015624, FBgn0034863, FBgn0011577, FBgn0040291, FBgn0035136, FBgn0040078, FBgn0026176, FBgn0000274, FBgn0259794, FBgn0003371, FBgn0002945, FBgn0011826, FBgn0004957, FBgn0023444, FBgn0042693, FBgn0025641, FBgn0003090, FBgn0034904, FBgn0000119, FBgn0029882	672	74	2054	0.950008044
KEGG	dme04330:Notch signaling pathway	7	0.179579	0.775984	FBgn0002643, FBgn0000524, FBgn0001169, FBgn0015805, FBgn0015624, FBgn0011591, FBgn0039734	672	22	2054	0.972537879
KEGG	dme04630:Jak-STAT signaling pathway	6	0.153925	0.796411	FBgn0010315, FBgn0041184, FBgn0003612, FBgn0020224, FBgn0015624, FBgn0014388	672	19	2054	0.965225564
KEGG	dme04013:MAPK signaling pathway	5	0.128271	0.88863	FBgn0000097, FBgn0004390, FBgn0000206, FBgn0003867, FBgn0003733	672	18	2054	0.849041005
KEGG	dme04070: Phosphatidylinositol signaling system	11	0.282196	0.907712	FBgn0085373, FBgn0036273, FBgn0085390, FBgn0034141, FBgn0085388, FBgn0003091, FBgn0010051, FBgn0034789, FBgn0037064, FBgn0015278, FBgn0000253	672	41	2054	0.820049361

KEGG	dme04350:TGF-beta signaling pathway	8	0.205233	0.96018	FBgn0026176, FBgn0015624, FBgn0034904, FBgn0024913, FBgn0020493, FBgn0011300, FBgn0034863, FBgn0040291, FBgn0035136	672	34	2054	0.719187675
KEGG	dme04150:mTOR signaling pathway	4	0.102617	0.997884	FBgn0010715, FBgn0260945, FBgn0053100, FBgn0037679, FBgn0035709	672	27	2054	0.452821869
PANTHER	P00025:Hedgehog signaling pathway	10	0.256542	0.43801	FBgn0046332, FBgn0250823, FBgn0015624, FBgn0003444, FBgn0003890, FBgn0003371, FBgn0001079, FBgn0024491, FBgn0052396, FBgn0004859	353	27	1160	1.217081104
PANTHER	P00057:Wnt signaling pathway	38	0.974859	0.513758	FBgn0010315, FBgn0002783, FBgn0015609, FBgn0011715, FBgn0015805, FBgn0020306, FBgn0065102, FBgn0003091, FBgn0250823, FBgn0000259, FBgn0015624, FBgn0000095, FBgn0260642, FBgn0022787, FBgn0004435, FBgn0000014, FBgn0046332, FBgn0259794, FBgn0039908, FBgn0003371, FBgn0025463, FBgn0011826, FBgn0010051, FBgn0003444, FBgn0000497, FBgn0027342, FBgn0011300, FBgn0042693, FBgn0023444, FBgn0010014, FBgn0001075, FBgn0259680, FBgn0036715, FBgn0032157, FBgn0043900, FBgn0040206, FBgn0030093, FBgn0035370, FBgn0011604, FBgn0039709	353	121	1160	1.032004308
PANTHER	P00038:JAK/STAT signaling pathway	2	0.051308	0.765127	FBgn0026160, FBgn0003612	353	4	1160	1.64305949

PANTHER	P00047:PDGF signaling pathway	16	0.410467	0.891568	FBgn0038588, FBgn0000567, FBgn0003091, FBgn0010051, FBgn0085447, FBgn0040068, FBgn0259680, FBgn0046332, FBgn0003969, FBgn0086901, FBgn0020412, FBgn0000274, FBgn0004390, FBgn0038043, FBgn0003371, FBgn0039796	353	62	1160	0.848030705
PANTHER	P00012:Cadherin signaling pathway	11	0.282196	0.931562	FBgn0015609, FBgn0000259, FBgn0003138, FBgn0011742, FBgn0027342, FBgn0000497, FBgn0001075, FBgn0036715, FBgn0046332, FBgn0003371, FBgn0039709	353	46	1160	0.78581106
PANTHER	P00021:FGF signaling pathway	12	0.30785	0.960153	FBgn0259680, FBgn0038744, FBgn0003969, FBgn0003091, FBgn0004390, FBgn0015763, FBgn0038973, FBgn0050060, FBgn0014388, FBgn0015278, FBgn0038972, FBgn0042693	353	53	1160	0.744026939
PANTHER	P00056:VEGF signaling pathway	4	0.102617	0.986284	FBgn0259680, FBgn0003091, FBgn0015278, FBgn0038603	353	23	1160	0.571498953
PANTHER	P00033:Insulin/IGF pathway-protein kinase B signaling cascade	6	0.153925	0.987576	FBgn0046332, FBgn0015396, FBgn0015278, FBgn0003371, FBgn0052006, FBgn0000659	353	33	1160	0.597476178
PANTHER	P00052:TGF-beta signaling pathway	10	0.256542	0.996187	FBgn0004885, FBgn0015624, FBgn0015396, FBgn0037930, FBgn0024913, FBgn0020493, FBgn0031461, FBgn0052006, FBgn0011300, FBgn0000659	353	56	1160	0.586806961
PANTHER	P00045:Notch signaling pathway	3	0.076963	0.999664	FBgn0002733, FBgn0011591, FBgn0039734	353	29	1160	0.339943343

1-Pathways with >=2 genes differentially expressed in Post-Eclosion AF
2-Discrepancy between "Count" and listed FBGN occurs when DAVID maps a probe to two different FBGN with the same DAVID gene name
