# Supplementary material for: Wnt Pathway Activation Increases Hypoxia Tolerance during Development
Source: PLoS One. 2014 Aug 5;9(8):e103292. doi: 10.1371/journal.pone.0103292 (PMC4122365; doi:10.1371/journal.pone.0103292)
Supplement: Table S4 — Wnt Pathway-Associated Genes Differentially Expressed in Post-Eclosion Hypoxia Tolerant Flies Grown at 4% O2. (PDF) [file pone.0103292.s008.pdf]

Table S4: Wnt Pathway-Associated Genes Differentially Expressed in Post-Eclosion Hypoxia Tolerant Flies Grown at 4% O2

FBgn[1]	Symbol	Enrichment Source	Wnt Pathway	Function	Affy Probeset	Mean Control	Fold-Change	p
FBgn0000014	abd-A	Panther	Canonical	Possible target gene	1637813_at	231.56	1.5667	5.42E-06
FBgn0000095	Antp	Panther	Canonical	Possible target gene	1640208_s_at	145.34	1.5141	3.30E-05
FBgn0260642	Antp	Panther	Canonical	Possible target gene	1624759_s_at	119.1	1.5018	5.55E-05
FBgn0000119	arr	KEGG, GO-BP	Canonical	Co-Receptor	1634211_at	211.76	1.3682	0.001352912
FBgn0015609	CadN	Panther		Cadherin	1633000_a_at	82.89	1.5962	6.88E-06
FBgn0004624	CaMKII	KEGG	Calcium	Kinase	1640534_s_at	375.61	1.4346	2.25E-04
FBgn0010014	CanB	Panther	Calcium	Dephosphorylates NFAT	1641460_at	524.45	1.5138	2.32E-05
FBgn0039908	CG11533/Asator	Panther	Canonical	Kinase	1627955_a_at	460.5	1.605	1.42E-06
FBgn0039282	CG11848/Bili	GO-BP	Canonical	Negative regulator	1640291_at	77.1	1.5769	1.42E-05
FBgn0034863	CG12227/skpF	KEGG	Canonical	β-catenin proteolysis	1639319_at	324.66	1.4468	1.64E-04
FBgn0034904	CG15800	KEGG	Canonical	β-catenin proteolysis	1624073_at	118.46	1.6831	2.42E-07
FBgn0039709	CG31009/Cad99C	Panther		Cadherin	1630399_at	192.22	1.5929	2.62E-06
FBgn0036715	CG6445/Cad74A	Panther		Cadherin	1635742_s_at	149.93	1.8881	1.67E-10
FBgn0042693	CG7913/PP2A-B'	KEGG, Panther	Canonical	Protein phosphatase, regulatory subunit	1628476_at	164.5	1.4113	4.99E-04
FBgn0026176	CG8881/skpB	KEGG	Canonical	β-catenin proteolysis	1636591_at	321.97	1.3734	0.001115791
FBgn0020309	crol	GO-BP	Canonical	Negative regulator	1626018_a_at	337.78	1.4093	4.45E-04
FBgn0025641	DAAM	KEGG	PCP	Actin binding	1629015_a_at	538.64	1.9488	9.59E-12
FBgn0011577	dally	KEGG, GO-BP	Canonical	Co-Receptor	1628489_at	929.18	1.482	5.74E-05
FBgn0015380	drl	GO-BP		Wnt binding protein	1624297_at	140.04	1.6969	1.23E-07
FBgn0000497	ds	Panther	PCP	Cadherin-related	1640627_at	100.8	1.7088	1.44E-07
FBgn0000658	fj	GO-BP	PCP	Wnt binding protein, protein kinase	1636091_at	98.27	1.4948	8.46E-05
FBgn0001075	ft	Panther, GO-BP		Cadherin-related	1624125_at	195.12	1.9448	1.65E-11

FBgn0004435	Galpha49B	Panther		GTPase	1639773_s_at	1586.75	1.7539	9.47E-09
FBgn0250823	gish	Panther, GO-BP	Canonical	Protein kinase	1626805_s_at	922.42	1.6424	4.10E-07
FBgn0046332	gskt	Panther	Canonical	Putative GSK3B homolog, β-catenin binding	1632217_at	283.67	1.5701	4.59E-06
FBgn0022787	Hel89B *	Panther		Helicase	1627970_at	295.96	1.5865	2.74E-06
FBgn0010051	Itp-r83A	Panther	Calcium	Inositol triphosphate receptor	1639461_a_at	101.1	1.4801	1.21E-04
FBgn0259896	nimC1	GO-BP		Wnt protein binding	1639502_at	304.1	1.5185	2.15E-05
FBgn0002945	nkd	KEGG, GO-BP	Canonical, PCP	Binds dsh	1630361_at	68	1.56	3.16E-05
FBgn0085432	pan	GO-BP	Canonical	Transcription factor	1629467_at	67.68	2.1253	7.14E-13
FBgn0003090	pk	KEGG	PCP	Interacts with Vang	1636612_a_at	89	2.3881	1.54E-17
FBgn0000274	Pka-C2	KEGG	Canonical	cAMP-dependent protein kinase	1632787_a_at	142.47	1.4964	5.52E-05
FBgn0003091	Pkc53E	KEGG, Panther	Calcium	Protein kinase C	1632478_a_at	192	1.5593	7.34E-06
FBgn0259680	Pkcdelta	Panther	Calcium	Protein kinase C	1628677_at	383.79	2.0954	4.75E-14
FBgn0040291	Roc1b	KEGG	Canonical	β-catenin proteolysis	1639817_at	214.85	1.533	1.54E-05
FBgn0020251	sfl	GO-BP	Canonical	Sulfotransferase	1631534_at	170.23	1.4608	1.33E-04
FBgn0003371	sgg	KEGG, Panther, GO-BP	Canonical	GSK3B homolog, β-catenin binding	1623084_at	193.58	1.7096	6.03E-08
FBgn0003371	sgg	KEGG, Panther, GO-BP	Canonical	GSK3B homolog, β-catenin binding	1630774_s_at	1131.65	1.5943	1.92E-06
FBgn0259794	sinah	KEGG, Panther	Canonical	β-catenin proteolysis	1638838_at	708.75	1.3782	9.50E-04
FBgn0029123	SoxN	GO-BP	Canonical	Chromatin DNA binding	1631408_at	162.67	2.0163	1.54E-12
FBgn0016977	spen	GO-BP	Canonical	Nucleic acid binding	1641518_a_at	833.94	1.4392	1.91E-04
FBgn0003866	tsh **	GO-BP		Transcription factor	1626150_at	121.64	1.5331	2.25E-05
FBgn0011300	babo	Panther	Canonical	Kinase	1623424_a_at	210.1	0.5842	3.53E-05

FBgn0025463	Bap60	Panther	Canonical	Brahma associated protein; Activator	1634157_at	306.34	0.5179	1.36E-06
FBgn0035370	CG1240	Panther	Canonical	Chromatin binding	1636036_at	639.65	0.5681	1.34E-05
FBgn0029882	CG3226	KEGG	Canonical	β-catenin proteolysis	1623702_at	669.89	0.6697	7.87E-04
FBgn0032157	CG5899/Etl1 *	Panther		Helicase	1624673_at	182.55	0.5165	1.74E-06
FBgn0030093	CG7055/dalao	Panther	Canonical	Chromatin binding	1631677_at	516.13	0.5839	2.76E-05
FBgn0000259	CkIIbeta	KEGG, Panther, GO-BP	Canonical	Protein kinase regulator	1637769_s_at	1221.23	0.6537	4.40E-04
FBgn0000259	CkIIbeta	KEGG, Panther, GO-BP	Canonical	Protein kinase regulator	1628344_at	118.53	0.404	9.15E-09
FBgn0010315	CycD	KEGG, Panther	Canonical	Cyclin-dependent protein kinase regulator; Target gene	1627295_s_at	212.13	0.5176	1.62E-06
FBgn0020306	dom	Panther	Canonical	Helicase	1633331_at	542.9	0.5932	4.12E-05
FBgn0020306	dom	Panther	Canonical	Helicase	1636034_at	276.23	0.3471	5.11E-11
FBgn0000524	dx	GO-BP		Protein binding	1626617_at	334.52	0.4934	3.68E-07
FBgn0023444	ebi	KEGG, Panther	Canonical	β-catenin proteolysis	1638600_at	268.11	0.5704	1.73E-05
FBgn0027342	fz4	Panther, GO-BP		Receptor for Wnt	1631777_a_at	212.88	0.5555	9.87E-06
FBgn0011604	Iswi	Panther	Canonical	Helicase	1641309_s_at	334.47	0.6721	8.98E-04
FBgn0040206	krz	Panther		β-Arrestin, binds to dsh	1623793_at	133.71	0.5872	5.81E-05
FBgn0002552	lin **	GO-BP		Catalytic activity	1623324_at	253.51	0.6672	7.99E-04
FBgn0002783	mor	Panther	Canonical	Chromatin binding	1640418_at	504.19	0.572	1.64E-05
FBgn0015624	nej	KEGG, Panther, GO-BP	Canonical	CREB binding protein	1622925_at	320.87	0.53	2.46E-06
FBgn0040078	pont	KEGG, GO-BP	Canonical	Helicase, transcription cofactor	1635279_at	442.56	0.5846	2.91E-05
FBgn0004957	por	KEGG, GO-BP	Canonical	Acyl transferase	1636527_at	80.47	0.6406	8.70E-04
FBgn0011826	Pp2B-14D	KEGG, Panther	Calcium	Dephosphorylates NFAT	1626781_at	575.74	0.6251	1.52E-04
FBgn0043900	pygo	Panther, GO-BP	Canonical	β-catenin nuclear targeting	1640730_at	433.99	0.6681	7.61E-04

FBgn0040075	rept **	GO-BP		Helicase	1630792_at	557.71	0.6441	3.16E-04
FBgn0087002	Rfabg	GO-BP		Retinoid and Fatty Acid Binding	1637843_at	5214.94	0.314	2.99E-12
FBgn0015805	Rpd3	Panther	Canonical	Histone deacetylase	1633700_at	363.93	0.5692	1.51E-05
FBgn0003415	skd	GO-BP	Canonical	Transcription cofactor; Positive regulator	1630269_s_at	118.27	0.44	6.92E-08
FBgn0003444	smo	Panther, GO-BP		G protein-coupled receptor activity	1634442_at	330.12	0.5642	1.23E-05
FBgn0011715	Snr1	Panther	Canonical	DNA binding	1625112_at	362.13	0.5896	3.73E-05
FBgn0086356	tum	GO-BP	Canonical	Small GTPase activator; Negative regulator	1634690_at	201.05	0.3178	9.59E-12

1- Affymetrix Probeset IDs converted to FlyBase genes using DAVID.
* Identified by DAVID; could not confirm in PANTHER
** Identified by DAVID; could not confirm in AMIGO
