# Supplementary material for: Wnt Pathway Activation Increases Hypoxia Tolerance during Development
Source: PLoS One. 2014 Aug 5;9(8):e103292. doi: 10.1371/journal.pone.0103292 (PMC4122365; doi:10.1371/journal.pone.0103292)
Supplement: Table S5 — Details of genetic experiments. Table S5A: Fly strains used in genetic cross experiments. Table S5B: Eclosion Rate Experimental Details. (PDF) [file pone.0103292.s009.pdf]

Table S5A: Fly strains used in genetic cross experiments.

Gene	Source	Stock / transformant number	Genotype	Chromosome(s) affected	Insertion chromosome(s)	Comments[1]
UAS Insert						
dsh	Bloomington	9453	w[*]; P{w[+mC]=UAS-dsh.myc}1-16	1;3	3	Expresses wild type dsh under UAS control
arm	Bloomington	4782	P{w[+mC]=UAS-arm.S10}C, y[1] w[1118]	1	1	Constitutively active
dally	Bloomington	5397	w[*] P{w[+mC]=UAS-dally.J}SJ1	1	1	
Rho1	Bloomington	7334	w[*]; P{w[+mC]=UAS-Rho1.Sph}2.1	1;3	3	Expresses wild type Rho1 under the control of UAS
bsk	Bloomington	6407	y[1] w[1118]; P{w[+mC]=UAS-bsk.A-Y}1	1;2	2	
rok	Bloomington	6669	y[1] w[*]; P{w[+mC]=UAS-rok.CAT}3.1	1;3	3	
CaMKII	Bloomington	29662	w[*]; P{w[+mC]=UAS-CaMKII.R3}2	1;2	2	Expresses the wild type R3 isoform of CaMKII under UAS control
RNAi-UAS Insert						
Apc	Bloomington	28582	y[1] v[1]; P{y[+t7.7] v[+t1.8]=TRiP.HM05070}attP2	1;3	3	Expresses dsRNA for RNAi of Apc (FBgn0015589) under UAS control, TRiP
sgg	Vienna	101538/KK			2	
nmo	Vienna	104885/KK			2	
arm	Bloomington	31305	y[1] v[1]; P{y[+t7.7] v[+t1.8]=TRiP.JF01252}attP2	1;3	3	Expresses dsRNA for RNAi of arm (FBgn0000117) under UAS control, TRiP.
arm	Bloomington	35004	y[1] sc[*] v[1]; P{y[+t7.7] v[+t1.8]=TRiP.HMS01414}attP2	1;3	3	Expresses dsRNA for RNAi of arm (FBgn0000117) under UAS control, TRiP.
dally	Bloomington	28747	y[1] v[1]; P{y[+t7.7] v[+t1.8]=TRiP.JF03175}attP2	1;3	3	Expresses dsRNA for RNAi of dally (FBgn0011577) under UAS control, TRiP.
dsh	Bloomington	31307	y[1] v[1]; P{y[+t7.7] v[+t1.8]=TRiP.JF01254}attP2	1;3	3	Expresses dsRNA for RNAi of dsh (FBgn0000499) under UAS control, TRiP.

Gal4 Insert						
elav	Bloomington	8760	w[*]; P{w[+mC]=GAL4-elav.L}3	1;3	3	Expresses GAL4 in the nervous system.
Hml	Bloomington	6396	w[1118]; P{w[+mC]=Hml-GAL4.G}6-4	1;2	2	GAL4 expressed in larval hemocytes; Hml normally expressed at low levels

1- Comments from Bloomington, except for UAS-arm, which was extracted from Flybase.

Table S5B: Eclosion Rate Experimental Details

		Eclosion Rate				
Targeted Tissue	Fly Strain	Mean	SD	# Expts	# Tubes	Total Pupae
Neuron						
5% O_2	elav-Gal4	0.014	0.026	4	12	469
	UAS-arm	0.108	0.07		12	983
	UAS-arm x elav-Gal4	0.541	0.195		12	973
	elav-Gal4	0.014	0.026	4	12	469
	UAS-dally	0.089	0.079		11	639
	UAS-dally x elav-Gal4	0.689	0.237		12	872
6% O_2	elav-Gal4	12.625	2.45982	1	8	408
	RNAi UAS-arm31305	35.46375	10.27307		8	471
	elav-Gal4 x RNAi UAS- arm31305	9.35	2.810694		8	530
	elav-Gal4	12.625	2.45982	1	8	408
	RNAi UAS-arm35004	36.8625	9.804655		8	493
	elav-Gal4 x RNAi UAS- arm35004	3.77	3.245995		8	458
	elav-Gal4	12.625	2.45982	1	8	408
	RNAi UAS-dally	24.55	12.877		8	476
	elav-Gal4 x RNAi UAS-dally	16.1375	6.046944		8	375
	elav-Gal4	12.625	2.45982	1	8	408
	RNAi UAS-dsh	31.37125	17.99768		8	526
	elav-Gal4 x RNAi UAS-dsh	8.215	3.980836		8	441
Larval Hemocyte						
5% O_2	Hml-Gal4	0.333	0.165	7	21	1161
	UAS-arm	0.141	0.118		21	1643
	UAS-arm x Hml-Gal4	0.754	0.16		21	2290

	Hml-Gal4	0.31	0.144	7	21	1173
	UAS-dally	0.099	0.077		19	917
	UAS-dally x Hml-Gal4	0.811	0.136		19	1164
	Hml-Gal4	0.358	0.174	5	15	712
	UAS-dsh	0.054	0.038		15	1207
	Hml-Gal4 x dsh	0.559	0.245		14	595
	Hml-Gal4	0.282	0.14	5	15	930
	RNAi UAS-sgg	0.048	0.056		15	705
	Hml-Gal4 x RNAi UAS-sgg	0.658	0.176		15	1092
	Hml-Gal4	0.294	0.178	3	9	524
	RNAi UAS-nmo	0.035	0.053		9	481
	Hml-Gal4 x RNAi UAS-nmo	0.652	0.23		7	388
	Hml-Gal4	0.263	0.136	3	9	452
	RNAi UAS-Apc	0.144	0.097		9	558
	Hml-Gal4 x RNAi UAS-Apc	0.448	0.176		8	357
	Hml-Gal4	0.299	0.079	3	9	536
	UAS-Rho1	0.172	0.131		9	632
	Hml-Gal4 x UAS-Rho1	0.855	0.093		9	724
	Hml-Gal4	0.332	0.122	3	9	492
	UAS-bsk	0.026	0.028		8	505
	Hml-Gal4 x UAS-bsk	0.829	0.064		9	709
	Hml-Gal4	0.313	0.088	3	9	579
	UAS-rok	0.014	0.022		9	334
	Hml-Gal4 x UAS-rok	0.217	0.189		9	675
	Hml-Gal4	0.294	0.126	4	12	616

	UAS-CaMKII	0.517	0.113		11	409
	Hml-Gal4 x UAS-CaMKII	0.865	0.141		12	836
6% O_2	Hml-Gal4	27.82875	5.096636	1	8	576
	RNAi UAS-arm31305	35.46375	10.27307		8	471
	Hml-Gal4 x RNAi UAS- arm31305	10.06875	3.388276		8	497
	Hml-Gal4	27.82875	5.096636	1	8	576
	RNAi UAS-arm35004	36.8625	9.804655		8	493
	Hml-Gal4 x RNAi UAS- arm35004	5.12	3.353744		8	368
	Hml-Gal4	27.82875	5.096636	1	8	576
	RNAi UAS-dally	24.55	12.877		8	476
	Hml-Gal4 x RNAi UAS-dally	12.19875	6.053576		8	537
	Hml-Gal4	27.82875	5.096636	1	8	576
	RNAi UAS-dsh	31.37125	17.99768		8	526
	Hml-Gal4 x RNAi UAS-dsh	10.65875	4.541512		8	476
