# Supplementary material for: Wnt Pathway Activation Increases Hypoxia Tolerance during Development
Source: PLoS One. 2014 Aug 5;9(8):e103292. doi: 10.1371/journal.pone.0103292 (PMC4122365; doi:10.1371/journal.pone.0103292)
Supplement: Text S1 — Supplementary Methods. (DOCX) [file pone.0103292.s010.docx]

**Supplementary Methods**

**STEM analysis.**

To identify processes that might have a genetic contribution but that were not identified by Vampire analysis to be shared by H and HR flies we explored the microarray data using Short Time-series Expression Miner (STEM) software[[1](#_ENREF_1)], treating the three developmental stages as a short time series. The algorithm clusters time-dependent expression profiles and allows for a comparison of two experimental groups with respect to profiles with shared genes. We compared H *vs* C, HR *vs* C and H *vs* HR, in each case using the excluded dataset as the denominator to generate the required ratio data input. We then manually evaluated the comparison figures with respect to Gene Ontology of genes whose direction of change differed at the post-eclosion data point. The results of this analysis, summarized in Figure S3C, suggest that reduced ribosome biogenesis and increased development-related gene expression in H flies depends on a hypoxic environment, whereas decreased DNA replication is at least partially genetic/epigenetic in origin.

**RT-PCR.**

First strand cDNA was synthesized using SuperScriptII reverse transcriptase and Oligo-(dT) primer. Real-time PCR amplification was performed using ABI Prism 7900HT Sequence Detection System (Applied Biosystems, Foster City, CA). For each reaction, 10 µl of 2× SYBR green PCR master mix (Applied Biosystems, Foster City, CA) and 0.5 µM of both forward and reverse primers along with 100 ng of each appropriate cDNA samples were mixed (total reaction volume: 20 µl). The relative expression level was calculated using 2−ΔΔCt method, as described previously[[2](#_ENREF_2),[3](#_ENREF_3)]. *Drosophila melanogaster* actin was used as internal control. The final results are presented both in terms of mean relative expression ± SD and as fold change of H or HR over C (Figure S3B). Three biological replicates for each condition (C, H, and HR) were tested in triplicate; technical replicates were averaged and used to calculate means and SD. Significance was determined using a two-sided t-test. The following primers were used:

| Oligo | Sequence (5'->3') |
| --- | --- |
|  |  |
| pim Forward | TGGAAAACAGATGCCTTCCGCCC |
| pim Reverse | TCAGGCTCTGCAGGCTCCATGT |
|  |  |
| MDR50 Forward | GCAGAAGGGAGTTTCGGCAGCC |
| MDR50 Reverse | ACACTCCCGGTCGGTCCACA |
|  |  |
| CKIIB Forward | TGCTCTTCATGGTGCATCCCGA |
| CKIIB Reverse | CTGCTGCCTGCAGCTGAATTTGAT |
|  |  |
| pk Forward | TCCGGACACACTGCCCCGAT |
| pk Reverse | TGTCCCGATCCCGCTCCCTC |
|  |  |
| CG13422 Forward | GCCGCCGATGTGGTCAGTTCA |
| CG13422 Reverse | GACTCCATGGTAACGCGCCGT |
|  |  |
| Act88F Forward | CCCAACAACCTCGGCTCGGAC |
| Act88F Reverse | TGAGCACCGACAACCGGAGGT |
|  |  |
| Act5C Forward | CTAACCTCGCCCTCTCCTCT |
| Act5C Reverse | GCAGCCAAGTGTGAGTGTGT |

**Hypoxia tolerance testing in adults.**

Adult recovery from anoxia: UAS-arm was crossed with Hml-Gal4 or elav-Gal4 in these experiments, and recovery time for each F1 was compared with its respective parental lines. Groups of 7-11 adult male flies (4-6 days old) were introduced into an airtight chamber that could be fed by room air (21% O_2_ ) or nitrogen (0% O_2_). After a 5-minute exposure to pure nitrogen, flies were reoxygenated and the time to first movement for each fly was recorded. Statistical significance was calculated using two-tailed unpaired Student t-test and data are presented as mean of all replicates ± 1.96 SEM (95% C.I.)

Adult activity in 2% O_2_: UAS-arm was crossed with Hml-Gal4 or elav-Gal4 in these experiments, which were done in sets consisting of one tube each of an F1 and its respective parents. Groups of 7-10 adult male flies (arm, Hml, arm X Hml: 5-6 days old; arm, elav, arm X elav: 8-9 days old) were placed in 15-ml glass tubes containing food and marked at 3 cm above the food line. Flies were gently tapped to the bottom of the tube and recorded as they climbed the wall. Flies were observed and recorded in both room air and under hypoxic conditions after a minimum acclimation of 2 hours at 2% O_2_. Under room air conditions, most flies in all groups climbed at least 3 cm in ≤2 seconds. Activity and climbing were greatly diminished in all groups after exposure to 2% O_2_.

1. Ernst J, Bar-Joseph Z (2006) STEM: a tool for the analysis of short time series gene expression data. BMC Bioinformatics 7: 191.

2. Ginzinger DG (2002) Gene quantification using real-time quantitative PCR: an emerging technology hits the mainstream. Exp Hematol 30: 503-512.

3. Livak KJ, Schmittgen TD (2001) Analysis of relative gene expression data using real-time quantitative PCR and the 2(-Delta Delta C(T)) Method. Methods 25: 402-408.
